# A Systematic Review and Qualitative Synthesis Resulting in a Typology of Elementary Classroom Movement Integration Interventions

**DOI:** 10.1186/s40798-019-0218-8

**Published:** 2020-01-06

**Authors:** Spyridoula Vazou, Collin A. Webster, Gregory Stewart, Priscila Candal, Cate A. Egan, Adam Pennell, Laura B. Russ

**Affiliations:** 1 Department of Kinesiology, Iowa State University, 534 Wallace Road, Ames, IA 50011 USA; 2 Department of Physical Education, University of South Carolina, 1300 Wheat Street, Columbia, SC 29208 USA; 3 Department of Physical Education and Exercise Science, Methodist University, 5400 Ramsey Street, Fayetteville, NC 28311 USA; 4 Department of Movement Studies, University of Idaho, 875 Perimeter Drive, Moscow, ID 83844 USA; 5 Natural Science Division, Pepperdine University, Malibu, CA USA

**Keywords:** Classroom physical activity, Activity break, Physical active lesson, Systematic review, Qualitative synthesis, Comprehensive school physical activity program

## Abstract

**Background/Objective:**

Movement integration (MI) involves infusing physical activity into normal classroom time. A wide range of MI interventions have succeeded in increasing children’s participation in physical activity. However, no previous research has attempted to unpack the various MI intervention approaches. Therefore, this study aimed to systematically review, qualitatively analyze, and develop a typology of MI interventions conducted in primary/elementary school settings.

**Subjects/Methods:**

Preferred Reporting Items for Systematic Reviews and Meta-Analyses (PRISMA) guidelines were followed to identify published MI interventions. Irrelevant records were removed first by title, then by abstract, and finally by full texts of articles, resulting in 72 studies being retained for qualitative analysis. A deductive approach, using previous MI research as an *a priori* analytic framework, alongside inductive techniques were used to analyze the data.

**Results:**

Four types of MI interventions were identified and labeled based on their design: student-driven, teacher-driven, researcher-teacher collaboration, and researcher-driven. Each type was further refined based on the MI strategies (movement breaks, active lessons, other: opening activity, transitions, reward, awareness), the level of intrapersonal and institutional support (training, resources), and the delivery (dose, intensity, type, fidelity). Nearly half of the interventions were researcher-driven, which may undermine the sustainability of MI as a routine practice by teachers in schools. An imbalance is evident on the MI strategies, with transitions, opening and awareness activities, and rewards being limitedly studied. Delivery should be further examined with a strong focus on reporting fidelity.

**Conclusions:**

There are distinct approaches that are most often employed to promote the use of MI and these approaches may often lack a minimum standard for reporting MI intervention details. This typology may be useful to effectively translate the evidence into practice in real-life settings to better understand and study MI interventions.

## Key Points

- This systematic review presents a typology of MI interventions based on their design, strategies, support, and delivery, to highlight the different types of existing MI interventions in the primary/elementary classroom setting.
- Nearly half of the interventions were researcher-driven, which may undermine the sustainability of MI as a routine practice by teachers in schools.
- There are distinct approaches that are most often employed to promote the use of MI and these approaches often lack a minimum standard for reporting MI intervention details.

## Background

Schools are viewed as natural settings to increase children’s physical activity because of their extensive access to youth (6–7 h per day, 36–49 h per week) and their existing infrastructure for physical activity promotion (teachers, facilities, and other resources) [1]. In collaboration with the Society of Health and Physical Educators (SHAPE) America, the Centers for Disease Control and Prevention issued recommendations to support the design, implementation, and evaluation of Comprehensive School Physical Activity Programs (CSPAPs) [2]. A CSPAP is commonly conceptualized as consisting of five components: (a) physical education, (b) physical activity during school, (c) physical activity before and after school, (d) staff involvement, and (e) family and community engagement [2]. Each component of a CSPAP can be designed to support children in developing the skills and knowledge needed for a physically active lifestyle and achieving the national recommendations of 60 min of daily physical activity [2].

Traditionally, school-based physical activity opportunities for children have been provided mainly through physical education and recess. However, academic and educational policy has led to school administrators cutting significant amounts of allotted time from these programs [3]. As a result, CSPAP components should be designed to expand children’s daily physical activity opportunities, as well as to reinforce physical education [4]. One approach in particular that has seen a significant rise in intervention focus is classroom movement integration (MI), which is the process of infusing movement, at any level of intensity, into regularly scheduled classroom time [5]. Examples of MI include providing movement breaks during academic lessons, teaching academic content through movement, and using regularly occurring transitions (e.g., between lessons) to increase movement opportunities [6].

Several review studies have been conducted to examine the effects of MI on students’ physical activity, as well as on students’ cognition, classroom performance, and academic outcomes [7–11]. Overall, the results of these reviews demonstrate that MI can be beneficial to students’ physical activity and academic achievement, and in the worst case, it does not decrease overall physical activity or interfere with school performance and/or academic achievement [7–11]. There seems to be sufficient evidence to support MI as educationally sound and potentially health-promoting [5]. The generally positive outcomes of MI interventions, along with the increase in the number of these interventions, underscore the need to ensure that the details of different intervention approaches are navigable and replicable, where appropriate, for researchers and practitioners. However, the specific nature of different MI strategies included in the design and implementation of published interventions has not been foregrounded in most reviews.

The most recent systematic review provided descriptive information about MI interventions in elementary/primary schools [11]. There was a wide range of intervention content reported across 39 studies with details such as the scheduled physical activity (type, intensity, duration, frequency), MI focus (academic or non-academic), and intervention dose (days per week; minutes per week). The authors also reported information about intervention fidelity, when such information was included in the reviewed studies. In tandem with these aspects of review [11], we aimed in the present study to build upon the still nascent descriptive knowledge base for MI interventions and provide a more in-depth qualitative analysis of the literature from nearly double the body of evidence. Specifically, our intention was to increase the transparency of varied intervention approaches for future consideration by intervention scientists, teachers, teacher educators, and others who may be interested in further testing specific MI strategies, adopting MI practices, and/or training school professionals to use and support MI. Toward this end, the purpose of this study was to systematically review and qualitatively synthesize MI interventions in elementary/primary schools. The goal was to, as thoroughly as possible, canvas all published MI interventions in the targeted setting so that we could distill the full scope of reported intervention details, thematically analyze, and subsequently classify different intervention approaches, resulting in a typology of MI interventions in primary schools.

## Methods

### Protocol

This study followed the Preferred Reporting Items of Systematic Reviews and Meta-Analyses (PRISMA) recommendations for systematic review reporting [12].

### Search Strategy

Studies were ascertained through a systematic search including four electronic databases (Google Scholar, PubMed, ERIC, PsycInfo) conducted from February 10 to March 31, 2017, by the third author. During this time period, the same author enrolled in a notification service for all four databases to ensure studies published during this timeframe would also be included. The second, third, and fifth authors identified a total of 14 keywords related to MI. These keywords were then divided into three categories: action, strategy, and participants (Table 1). Researchers conducted pilot searches consisting of all possible combinations of the 14 keywords and identified 11 search combinations that were found to elicit the most relevant study results (Table 1). A separate search was conducted for each of the 11 keyword combinations in each of the four databases (totaling 44 searches). The default “AND” was used between keywords in each combination (e.g., exercise AND int* AND class*). Searches were sorted by relevance and restricted to records published in English. No other restrictions were used (e.g., date range). In most cases, relevant records appeared within approximately the first 200 records returned for each search. However, to ensure no relevant records were omitted, the first 2000 returned records from each search were exported into an Excel spreadsheet to begin the identification stage of PRISMA [12].

**Table 1:** Databases searched, keywords by category, and keyword combinations used for the review

Databases	Keyword category 1: action	Keyword category 2: strategy	Keyword category 3: participants
PubMed	Physical activity	int*	Child*
Google Scholar	Exercise	Trial	Youth
ERIC	Movement	Program*	Student*
PsycINFO		Training	Class*
			Elementary
			Primary
			School

Keyword combinations used for the review

exercise AND int* AND class*

physical activity AND trial AND class*

movement AND program* AND class*

exercise AND int* AND school

physical activity AND trial AND school

movement AND program* AND school

exercise AND int* AND school

physical activity AND trial AND elementary

movement AND program* AND elementary

physical activity AND class* AND child*

physical activity AND child* AND school*

### Inclusion and Exclusion Criteria

The following set of inclusion criteria was utilized to select papers for this qualitative synthesis:

1. Intervention study design in a school setting.
2. At least 1 component of the MI intervention had to take place in the regular classroom setting, at a regular classroom time.
3. Study population included primary/elementary school age children (i.e., 4–10 years old; Kindergarten—5th Grade). Studies were included if they contained pre-school age (i.e., 3–4 years old) or middle school age children (i.e., 10–13 years old) in the sample population but were excluded if the entire population consisted of those populations.
4. Presented original data with results. Dissertations, theses, review studies, and conference presentations/proceedings were excluded.
5. The university database had access to the study.

### Study Selection

**Fig. 1:**
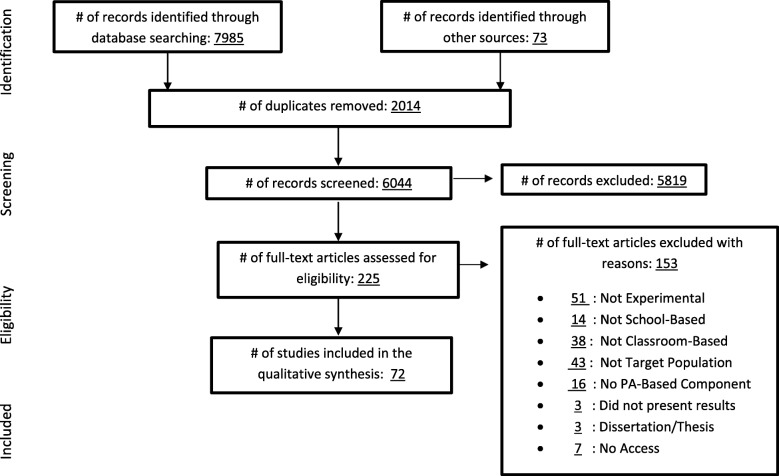
PRISMA flow diagram showing flow of studies through the review process

A systematic search generated 7985 possible records in the initial search, and 73 possible records were identified through other sources, meaning previous reviews and meta-analyses of this literature and extensive search of reference lists from obtained articles (Fig. 1). After using Excel to eliminate duplicates (*n* = 2014), the titles and abstracts of 6044 were screened by the first, third, and fifth authors. A total of 225 publications were identified as possibly relevant to the inclusion criteria, and the earlier mentioned authors reviewed these records in full text. Disagreements between reviewers regarding inclusion/exclusion of a study were resolved through discussion. Of the 225 full-text articles reviewed, a total of 72 articles satisfied the inclusion criteria and were included in the subsequent phases of the review.

### Data Extraction

All authors extracted relevant data for the analysis from the included articles, and the first author reviewed all records from the original studies and the information included in the review to ensure the quality and accuracy of the data extracted. Data extraction from each study included country, school location (e.g., urban, rural, suburban), participant characteristics (e.g., grade levels and number of children that received the intervention), intervention characteristics (e.g., intervention design, intervention strategy, description of program, physical activity characteristics, resources and equipment, training), and implementation characteristics (e.g., implementation fidelity, implementation measures) (Additional file 1).

### Data Analysis

Before analyzing the data, the first three authors collapsed the information extracted from the included articles into categories, based on its different areas of general focus (e.g., participant characteristics, school characteristics, intervention characteristics). Subsequently, these researchers used both deductive and inductive data analysis techniques. The deductive approach consisted of drawing upon two previous studies [6, 13] to analyze the data within and across categories. In the first study [6], an observation system that codifies MI into distinct strategies (e.g., movement break, opening activity, transition) was developed. In the second study [13], common facilitators (e.g., administrative support, availability of resources) and barriers to MI (e.g., lack of time, lack of resources) in elementary school classrooms were identified using a social-ecological perspective, which considered variables that could be associated with MI at multiple levels of influence (e.g., intrapersonal, interpersonal, institutional). The results from these previous studies served as an a priori analytic framework for the present investigation, providing the researchers with existing, relevant, and evidence-based lenses to search for common and distinguishing features among the various MI intervention approaches.

Modified analytic induction [14] was used to incorporate inductive techniques into the data analysis. While comparing the data to the a priori framework and considering features of each intervention in light of MI strategies, facilitators, and barriers, the researchers also searched for intervention features that the a priori framework did not help to classify. These features (e.g., who planned/implemented the intervention strategies; the intensity of the physical activities used in the intervention) were then examined to find commonalities and consistencies. The researchers recursively analyzed the data using these deductive and inductive techniques until they felt that each intervention could be classified into a unique “type,” and that the different types of interventions (comprising what we refer to as a “typology”) parsimoniously captured the diverse range of MI intervention approaches.

Movement break is defined as a physical activity in the classroom that does not include academic content and it is used as an activity break, whereas, academically infused, or integrated MI refers to any physical activity that is used to review or teach academic content. Opening activity MI is movement directed by the teacher within the first 10 min of the official start of the school day. Transitional MI (both teacher-directed and non-teacher-directed) involves students walking from point A to point B. Reward/incentive MI is a movement provided by the teacher as an obvious reward for providing a correct response or behavior in class [6].

## Results

A total of 72 MI interventions [15–86] were classified based on their approach into 4 MI categories according to their design: student-driven, teacher-driven, researcher-teacher collaboration, and researcher-driven interventions. These categories were mainly informed by the inductive analysis, as they represent new perspectives not reported in the previous studies (i.e., the a priori analytic framework) used for the deductive analysis. Next, each unique type of intervention is described, along with three other grouping categories: (a) the adopted strategies (movement break, academically infused, opening activity, transition, reward), informed by the a priori framework, (b) the level of support received at an institutional and intrapersonal level (resources and training), also informed by the a priori framework, and (c) the characteristics of the delivery (dose, intensity, type of physical activity, and fidelity), informed by the inductive analysis.

**Fig. 2:**
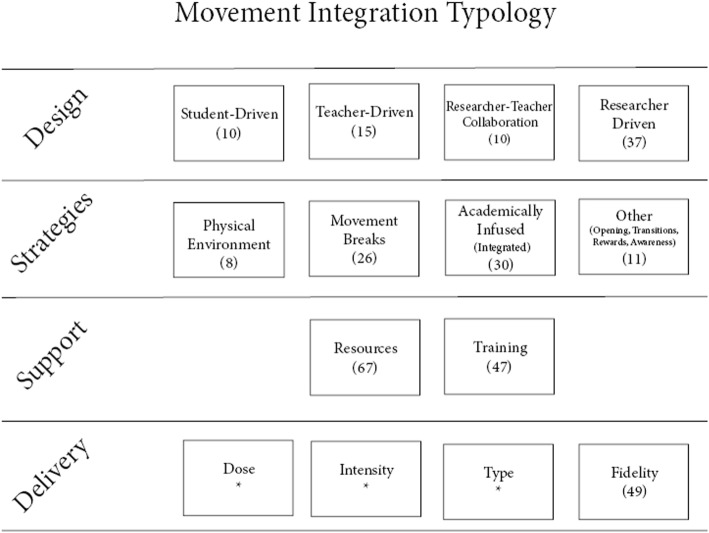
Typology of MI interventions. *Note.* Numbers in parenthesis represent the overall number of interventions for each category. *Frequency table is provided separately for those categories

The categories of the MI intervention approach and the total number of studies in each category are presented in Fig. 2, whereas the number of interventions for the different categories is presented in Table 2. The focus of the support given to teachers to promote their use of MI can be conceptualized in different ways. One way to understand support for MI is to consider existing facilitators and barriers to its use [13]. According to a previous systematic review, facilitators and barriers can be separated into two levels of influence on MI: institutional (factors within the school environment that are beyond the teacher’s direct control) and intrapersonal (factors specific to the teacher’s background, experience, and beliefs). At the institutional level, facilitators included administrative support and availability of resources, whereas barriers included lack of time, lack of resources, lack of space, and lack of administrative support. At the intrapersonal level, facilitators included a perception that physical activity is valuable, perceived ease of implementation, and teacher confidence, whereas barriers included implementation challenges, lack of teacher motivation, and lack of training [13].

**Table 2:** Frequency of interventions based on design and overall data on strategies, support, and delivery

	Student-Driven (10)	Teacher-Driven (15)	R-T Collaboration (10)	Researcher-Driven (37)	Total (72)
Strategies^*#*^					
Physical environment	8	0	0	0	8
Academically infused	0	6	7	17	30
Movement break	3	8	3	10	24
Other	1	3	0	7	11
Support: training					
Yes, by Researcher/Experts (before impl.)	2	5	8	17	32
Yes, by School Staff/Adm. (before impl.)	1	4	0	3	8
Yes, but unclear by whom	0	1	1	0	2
Ongoing (after impl.)*	1	6	2	5	14
Not required/provided	3	1	1	13	18
Not reported/missing	4	4	0	4	12
Support: duration of training					
< 1 h	1	0	1	1	3
Up to a school day (between 6 and 8 h)	0	4	3	4	11
> a school day	0	1	1	0	2
Not required/provided	3	1	2	5	11
Not reported/missing	6	9	3	27	45
Support: resources					
Desk/chairs/stability balls	9	0	0	0	9
Fitness equipment	1	5	0	2	8
Lessons plans/cards/manuals	1	4	9	18	32
Websites/electronic/DVD	0	2	5	5	12
Not required	1	4	1	6	12
Not reported/missing	0	4	0	9	13
Delivery: intensity/type of PA#					
Light	8	0	0	6	14
MVPA	2	9	9	26	42
FMS	0	8	1	2	11
Fitness	1	0	3	3	7
Dance	0	4	1	1	6
Yoga/breath/stretch	1	3	2	6	12
Other	1	0	0	3	4
Not reported/missing	0	0	0	4	4
Dose per day					
< 10 min	0	4	5	5	14
10–20 min	0	5	2	17	24
21–35 min	2	1	2	7	12
> 35 min (50–90 min)	2	3	0	2	7
Throughout the day (no specific dose)	6	0	0	0	6
Not reported/missing	0	2	1	6	9
Duration of intervention					
Acute (< 1 week)	2	1	0	8	11
2–3 weeks	0	1	0	0	1
1–6 months	3	4	3	13	23
7–11 months	5	5	3	5	18
1–3 years	0	1	2	8	11
Not reported/missing	0	3	1	0	4
Fidelity					
Observation	4	1	1	5	11
Teacher log	0	4	3	7	14
Activity monitor	1	1	0	3	5
Combination/other	2	1	4	8	15
Not reported/missing	3	8	2	14	27

^#^as assessed by the researchers based on content provided; *independent of other categories; *Impl.*, implementation; *MVPA*, moderate-to-vigorous physical activity; *FMS*, fundamental motor skills

The majority of the interventions had a research-driven design, were mainly focused on movement breaks and academically infused activities, provided some kind of resources and training to the participating teachers or schools, and measured implementation fidelity. The dose (duration per day in minutes and/or frequency per week) for MI varied from 2 to 90 min per day with the most frequent being under 10 min. The duration of the intervention varied from a day or a week (mostly in acute studies) to 3 years with the most frequent being from 1 to 6 months. The MI activities were mainly moderate-to-vigorous intensity and the type varied greatly among fundamental motor skills, fitness aerobic, fitness resistance, yoga, stretching, and dance. The missing information for delivery was high for all categories with the highest being on the type of physical activities used for MI (26 studies) and the implementation fidelity (23 studies). A total of 19 MI interventions were part of a multi-component approach that targeted multiple behaviors (e.g., nutrition, health education) and/or multiple contexts (e.g., classroom, PE, family). All data presented in the results section are also provided collectively in Additional file 1 along with the information regarding the study population.

### Student-Driven

A total of 10 interventions [15–24] were classified as student-driven MI. These interventions were characterized by high student autonomy in adding movement in the classroom throughout the school day, mainly by standing or using alternative options to sitting, without teacher prompts and without interrupting the teacher during instruction. The majority of student-driven interventions [15–21, 23] were centered on changes in the physical environment, which is defined as when the “equipment used is facilitative, resulting in student activity, regardless of level of intensity” (p. 301) [6].

Student-driven MI interventions utilized ergonomic furniture, including stand-based/height-adjustable desks [15, 16, 18, 19, 23] and stability/therapy balls [17, 20, 21, 23] or a combination of ergonomic furniture and room organization (e.g., strategically placing materials on different sides of the classroom) [18]. The remaining two student-driven interventions provided autonomy for the students to engage in movement breaks based on their preference. The first study used fitness stations in the classroom where the students received incentives depending on how frequently they used the stations [22], and the second study promoted self-directed physical activity outside of school after a short introduction of a variety of activities at school [24]. In student-driven MI interventions, researchers either provided schools with ergonomic furniture or assisted the school in writing a grant to help acquire the furniture.

Most student-driven MI interventions did not include [15, 16, 22] or report [17, 18, 21, 23] a teacher training. Interventions that did include a training [19, 20, 24] focused on appropriate use of the desks or nutritional education [22]; one of the interventions included pedagogical strategies for reducing sitting time and adapting traditional delivery of the academic curriculum [19]. When reported, training was delivered by a physical education teacher in collaboration with a researcher [20] or the County Health Coordinator [24] and in one intervention training lasted for 30 min [19].

Regarding delivery, the intensity of the physical activities of most student-driven interventions was light [15–21, 23], such as standing, dynamic sitting, and upper body movements (rotation, lateral flexion); one incorporated a manipulative motor skill focus by throwing bean bags while spelling [19]. Two interventions incorporated higher intensity levels of physical activity (i.e., moderate-to-vigorous physical activity [MVPA]) [22, 24]. The majority of student-driven interventions included environmental changes that were available in the classroom throughout the school day [15, 16, 20–23] and in most cases throughout the school year [15, 16, 19, 21, 22]. Therefore, a specific dose of the MI was not provided with the exception of four interventions [17–19, 24] that recommended MI for 30–60 min per day, with two of them being acute studies [17, 18]. The most common measure for intervention fidelity was direct observation [17, 20, 21, 23]. Three interventions did not report information about fidelity [15, 16, 24] whereas the remaining interventions used a questionnaire [18], activity monitors [19], or personal meetings with the teachers [22]. Four interventions delivered the program as intended [16, 18, 20, 23].

### Teacher-Driven

A total of 15 interventions [25–39] were classified as teacher-driven MI interventions. These interventions were characterized by high teacher autonomy in the design and implementation of the movement opportunities. Teacher-driven MI included mainly academically infused (integrated) movement [29, 32, 35, 36, 38, 39] and movement breaks [25–28, 30, 31, 34, 37]. A limited number of interventions incorporated movement as an opening activity for the school day in addition to the movement breaks [28, 37], or a reward for classroom behavior [28]. Information about the MI strategy was missing from one intervention [33].

The top three most used resources and equipment in teacher-driven interventions were as follows: fitness equipment such as jump ropes, exercise bands, bean bags, and sport balls [28, 32, 34, 36, 37], written materials or material kits such as lesson plans, activities, and handouts [27, 28, 34, 36], and exercise videos [34, 37]. Four interventions encouraged teachers to use MI but did not require the use of any resources or materials [26*, 29, 35, 38] and four interventions did not report that information [25, 30, 31, 33].

The majority (10 out of 15; 6 being academically infused MI) of the teacher-driven interventions offered training [27–30, 32, 34–36, 38, 39] delivered by researchers [32, 34, 36] or PE/Health teachers and school coordinators [27, 30, 39]. The training lasted from 3 h [35] to 20 h [30] with the majority of the interventions that reported the duration of the training being a full school day [29, 34, 36]. Additional ongoing support throughout the implementation included meetings and workshops delivered by school staff (e.g., PE teachers) [27, 28, 34, 35] and emails or use of a website to share lesson plans provided by the research team [32, 36].

Most of the MI activities focused on MVPA levels [26–29, 32–34, 37, 39], followed by fundamental motor skills [26, 29, 31, 32, 34–37], whereas some interventions incorporated other types of physical activities, such as dance [34–36, 38], stretching [26, 38], and yoga [38]. None of the physical activities was light intensity, compared with the previous section of student-driven interventions where the majority were light physical activity. About an equal number of interventions focused on MVPA levels and/or fundamental motor skills during movement breaks and academically infused movement. Over half of the teacher-driven MI activities were below 20 min in duration [25–27, 29, 31, 33, 34, 37, 38]; three of the MI activities ranged from 30 to 60 min [32, 35, 36], one was more than 60 min per MI activity [39] and two interventions did not report on activity duration [28, 30]. Almost half of the interventions had a typical weekly frequency of 2–5 times [25, 29, 32, 34–37] with two interventions provided flexibility for teachers to implement the activities as needed [38, 39], whereas six interventions did not report the typical frequency of MI per week [27, 28, 30, 31, 33, 36]. One intervention was an acute study with 1-week delivery dose [25]. Six out of the 15 teacher-driven interventions measured fidelity with the most common measure being a teacher log [28, 34, 36, 38] followed by direct observation [35] and activity monitors [33]. Three interventions delivered the programs as intended [28, 34, 36].

### Researcher-Teacher Collaboration

A total of 10 interventions were classified as researcher-teacher collaboration MI interventions [34–49]. Researcher-teacher collaboration MI interventions were characterized by the design and implementation of physical activity opportunities as a collaborative effort between researchers and teachers. This collaboration was achieved through the researcher designing physical activity opportunities and then allowing the teachers to adapt these activities to provide a “better fit” for their individual classrooms [45, 48], providing teachers with a menu of activities to select from or when and how to use them [42, 43, 47, 49], or working together (researchers and teachers) to create lesson plans [40, 41, 44, 46]. The majority of the researcher-teacher collaboration interventions incorporated academically infused movement [40–42, 45–49] with only three focusing on movement breaks [43, 44, 49].

All researcher-teacher collaboration interventions provided materials/equipment in the form of lessons plans, a teacher guide or a fitness manual [40–42, 44–46], or activity cards, games, and DVD [43, 48, 49]. However, none of these interventions provided fitness equipment to the teachers, unlike the student-driven and teacher-driven interventions. All but one intervention [48] provided training before the start of the implementation period, delivered by researchers [41–47, 49], with the duration varying from 30-min individual meetings [45] to two full school days [41]. Throughout the implementation, only two researcher-teacher collaboration interventions [45, 47] provided ongoing support with one of them included two email/phone communications with the participating schools [47].

Nine of the 10 researcher-teacher collaboration interventions focused on MVPA levels [40–45, 47–49], whereas only two incorporated other forms of physical activities like dancing, yoga and stretching [42], or fundamental motor skills [48]. Most research-teacher collaboration interventions mentioned the dose of their MI activities; five of the interventions reported MI activities of 10 min or less [40, 42, 43, 48, 49], four had MI activities between 20 and 30 min [41, 44, 45, 47] with only one intervention not reporting the duration of MI per day [46]. Half of the research-teacher collaboration interventions reported a weekly frequency of 2–5 times [41–44, 47, 48], while two interventions allowed teacher discretion and flexibility to incorporate the MI activities [40, 49] and two did not report weekly frequency [45, 46]. The most common measure for intervention fidelity was a teacher log [43, 47, 48], in combination with direct observations [40, 41, 49], as well as focus groups and activity monitors [45]. One study used only direct observations [42] and two studies did not report fidelity [44, 46]. Three research-teacher collaboration interventions delivered the program as intended [42, 45, 47].

### Researcher-Driven

A total of 37 interventions were classified as researcher-driven MI interventions [50–86]. In these interventions, the researchers controlled the design and/or implementation of physical activity opportunities. The participating teachers were usually responsible for delivering the intervention as designed by the researcher. Like teacher-driven MI interventions, researcher-driven interventions included both academically infused movement [50–52, 54–56, 58–62, 70, 71, 74, 79, 85, 86] and movement breaks [53, 64–66, 68, 73, 75, 80, 81, 83]. Some research-driven interventions included MI programs that incorporated a body awareness approach, in which body posture, coordination, mindful movements, breathing, and relaxation were the main focus [57, 72, 76, 77]. Two interventions integrated movement as an opening activity or during school transitions [72, 73] while four studies did not report how movement was integrated in their intervention [63, 67, 69, 78].

The majority (21 out of 37) of the researcher-driven interventions provided written materials in form of lesson plans, manuals or resources, such as CDs, magazines, activity cards, and kits [50–55, 57–59, 61, 70–72, 74, 75, 77, 79, 80, 82, 83, 85, 86] while six interventions did not share resources with teachers, schools, and/or students [56, 62, 65, 68, 73, 76]. The remaining nine interventions did not report any information about resources [60, 63, 64, 66, 67, 69, 78, 81, 84]. In addition to the aforementioned resources, only two studies utilized equipment (e.g., sports balls) in their programs [57, 85].

Seventeen interventions provided training to classroom teachers delivered by researchers [50, 51, 54–61, 70, 72–74, 78, 85, 86] and six interventions identified professionals such as qualified physical education teacher [66], trained intervention coaches [69, 71], the researchers [62, 65, 80], or trained medical students [81] to deliver the intervention instead of training the classroom teachers to deliver MI. Two interventions used a standardized video for MI [77, 83]. Seven researcher-driven interventions did not provide training [53, 63, 64, 67, 68, 75, 79] and four did not report if there was any training [52, 76, 82, 84]. The duration of the training varied from 30 min [54] to a full school day [50, 60, 61, 78]. Ongoing support throughout the implementation was reported only by five interventions [51, 58, 60, 61, 86] with one providing weekly consultations [51] and two offering 1–2 booster sessions halfway through [60, 61]. The remaining two interventions reported providing consultation support throughout implementation without being specific about the type of support.

The majority of the researcher-driven interventions (26 out of 37) focused on the intensity (MVPA) of the MI activities [52–54, 56–59, 61–66, 68, 69, 71–75, 79–84, 86], whereas some had a fitness approach, either in the form of aerobic exercise [81, 83] or strengthening exercise [83]. Two researcher-driven interventions focused on fundamental motor skill development [78, 85] and six on light intensity physical activities, such as stretching, yoga, coordination and breathing exercises [57, 72, 76, 77, 84, 86]. Lastly, five interventions did not report the type and/or intensity of the activities [51, 55, 60, 67, 70]. The duration of MI activities per day varied substantially from under 10 min [52, 65, 68, 73, 76] to over 50 min [60, 66], with the most common duration being 10–20 min [51, 53, 56, 58, 59, 61–65, 70–72, 75, 80, 83, 85, 86] and the shortest being 3–4 min [68, 73]. Six studies did not specify the recommended duration of the activities [50, 55, 57, 67, 69, 84] and seven lasted between 20 and 30 min per day [54, 74, 77–79, 81, 82]. Almost all of the researcher-driven interventions were recommended on a daily basis or did not provide information about weekly frequency.

About one-fourth of the researcher-driven interventions did not measure fidelity [52, 53, 63, 64, 67, 69, 72, 76, 82, 84], and of those that measured it, about half studies reported that the intervention was delivered as intended [50, 51, 58, 61, 62, 65, 68, 70, 74, 80, 81, 83, 85]. The measures used for fidelity varied between teacher logs [54–56, 58, 73, 75, 77], direct observation [51, 57, 65, 68, 74], activity monitors [62, 70, 80], meetings or ending surveys [60, 86], or a combination of measures [50, 59, 61, 66, 78, 79]. 

## Discussion

As demonstrated in this paper, in recent years, this line of research has shown an increase in the rate of accumulation of data on MI interventions. Despite the developing empirical studies, little research has examined the nature of classroom MI interventions, which makes it difficult to trace and unpack the various intervention approaches that have been used in previous studies. This, in turn, exacerbates efforts to compare or replicate previous MI interventions and poses a challenge to evaluate the feasibility and sustainability of different approaches to increasing MI. In the present study, a systematic literature search ascertained a total of 72 studies that reported MI interventions with primary-/elementary-age children. Using the information provided in these studies, and drawing from previous MI research [6, 13] as an a priori framework for analysis, the researchers created a typology of MI interventions based on their design, the implemented strategies, the level of institutional and intrapersonal support, and the type of delivery.

### Design

The design of the MI interventions (student-driven, teacher-driven, researcher-teacher collaboration, and researcher-driven) was a unique factor that emerged from our qualitative analysis that had not been identified in previous reviews on MI [11] or studies that examined implementation factors of MI [87–89]. MI interventions in this review were found to be more researcher-driven than teacher-driven, student-driven, or collaborative in nature. The majority (37 studies) of the interventions involved the researcher as a key stakeholder in intervention design and implementation. As the results demonstrated, in researcher-driven interventions, training was provided to the teachers mainly before the start of the intervention, whereas the more teachers involved in the design of the activities, the more administrative support was provided throughout the implementation period (13.5% in researcher-driven, 20% in researcher-teacher collaboration, and 40% in teacher-driven interventions). Researcher-driven interventions have the potential to be limited in scope due to a lack of first-hand/insider knowledge about the particular school context(s) and influencing variables. Researcher-driven interventions also may lack sustainability based on a lack of teacher buy-in due to academic/teaching barriers and intrapersonal factors, such as lack of motivation, perceived value for the MI, and perceived competence [13, 87, 88]. It is essential for a research to become more pragmatic and translational with programs accounting for the constraints that the schools have, such as lack of time. Interventions with a higher involvement of teachers on the design of the programs may be more pragmatic and potentially more sustainable. As previous research has shown, teachers are often implementing MI in the classroom and are interested in doing more when receiving support and ideas as well as learning more about the research on MI [89].

A limited number of interventions had a collaborative approach between researchers and teachers. A community-based participatory research (CBPR) approach may benefit MI interventions [90]. CBPR involves collaboration between change agents in the school community (e.g., classroom teachers, administrators, physical education teachers) and researchers throughout all phases of the research process [91]. Health promotion research provides strong evidence that CBPR can lead to greater implementation efficiency, reduce dependency on researchers, ensure cultural and local sensitivity, enhance program productivity, promote equitable distribution of services, and increase program sustainability [92]. Ultimately, partnerships through CBPR take advantage of stakeholders’ localized knowledge as well as researchers’ empirical knowledge and scientific expertise to promote a more contextually valid intervention design [92]. The results of the review demonstrated that the number of interventions that were developed by teachers or provided the autonomy to students to benefit from the MI when needed was high (35%). It can be assumed that many of those MI interventions were easy to implement as they did not require training but relied on the confidence and motivation of the participating teachers. We believe that these types of interventions hold promise for long-term implementation and sustainability on MI as teachers and students can be more independent and implementation can rely more on autonomous motivation without being driven by external factors (e.g., because of requests from the principal or the research team) [93]. However, it should be emphasized that institutional support, regarding equipment, and space were critical for the implementation of the student-driven MI interventions, whereas the need for equipment or resources in the teacher-driven interventions was low to zero and, in most cases, limited to small fitness equipment, like stretching bands or balls.

### Strategies

The present study demonstrated that, overall, 75% of MI interventions (54 studies) in this review implemented movement as a break from instruction or in the form of integrated lessons (academically infused), strategies that have been classified as “other movement” in previous MI interventions [6]. Interestingly, the majority (70%) of the researcher-teacher collaboration MI interventions and about half (46%) of the researcher-driven MI interventions were focused on academically infused physical activities. Even though the existing evidence is limited to draw clear conclusions about the effectiveness of integrated physical activities on academic outcomes [94], multiple advantages have been identified in recent studies (e.g., directly facilitating learning, increasing intrinsic motivation for the educational process) [48, 95, 96]. Arguably, an additional major benefit may be that it can further allay concerns that the time spent on physical activity is a time taken away from academics, especially since lack of time is a common barrier identified by teachers [13, 87, 88]. However, achieving true integration can be a labor-intensive task requiring collaborative teams of activity and education experts that may not be feasible in many cases. More research is needed to shed light on the required delivery attributes of academically infused lessons and the characteristics of the teachers to successfully implement this strategy in the classroom.

Fewer interventions focused on manipulating the physical environment (e.g., pedal desks) or on strategies such as transitions, opening activities, and rewards. A unique category that emerged in our study was the “awareness” strategy that included bodily awareness and mindful control for correct posture, elimination of noise in the classroom, and/or increased concentration. Considering the unequal number of interventions on the different MI strategies (mainly activity breaks and academically infused), future studies should expand on MI strategies that have received less attention, such as transitions, opening activities, awareness activities, and rewards. It is expected that transitions hold promise on being easily implemented in the classroom due to the fact that transitions are a naturally occurring and often frequent part of classroom routines that require minimal resources and time to promote student physical activity [6]. Considering that the aforementioned MI strategies vary substantially in their content and structure, it is crucial for researchers to identify or systematically examine factors that could be perceived as barriers and facilitators for each of the MI strategies separately. For example, academically infused physical activities may require more experience in teaching an academic concept, whereas an opening or a transition activity may require more skills in managing space and time. Future research is needed in order to better understand how physical activity can be implemented through a variety of MI strategies in the academic classroom and design meaningful programs that will defeat the impression that movement and learning are pitched against each other in an antagonistic relation.

### Support

Overall, the majority of interventions (67 studies) reviewed in the present study primarily focused on providing resources (e.g., stability balls, resource manuals; equipment bins), which represent institutional factors for implementation. Many of these resources were prepacked material that can be easily provided and are cost efficient. These materials usually came in the form of a resource manual with example activities for teachers to choose from or a step-by-step curriculum guide for teachers to implement. However, descriptions of these materials generally lacked detail. At a minimum, studies should provide examples of the materials/activities or a link to the materials/activities. Designing programs that require minimum resources may be considered more feasible for implementation. Cost-effectiveness studies are needed in order to better understand the efficacy and feasibility of MI programs.

Many of the MI interventions in this review (42 studies) provided training of some type to the teachers or school staff as an intervention component. However, the information provided about these trainings was limited. Details about trainings typically were presented in 3–4 sentences identifying parts of the following information (rarely all of the categories presented here were reported by one study): the rationale for the training, the purpose of the training, the length of the training, who led the training, and a broad outline of what the training included. Little to no information was provided with respect to the theoretical/empirical basis of the training; specific training objectives; training activities or professional learning experiences; teacher satisfaction with the training; and steps taken to determine if the training achieved the desired professional development for the intervention to be feasible or successful. Training was typically provided before the start of the intervention and lasted from a couple of hours to one or two full days.

Ongoing support and feedback throughout the intervention period were rarely provided, whereas in most interventions, the level of support or the climate from the school administrators was not measured. A possible solution to ongoing support for MI interventions could be the use of online communities of practice as a platform for interactions among teachers with a shared goal. Online communities of practice have been proposed as a promising avenue for teachers to share their best practices and experiences for MI and to strengthen their confidence through increased awareness/knowledge and peer modeling [90, 97, 98]. However, as this strategy is relatively new, understanding how to best engage teachers in an online community of practice by overcoming barriers such as lack of time remains unclear [99]. Despite the support at the institutional level, an ecological model of behavior change emphasizes intrapersonal variables as central and most directly influential to changing the behavior of targeted individuals (e.g., teachers) [100]. MI interventions that only focus on institutional factors related to MI do not empower teachers to use MI and may lack sustainability once the institutional resources are no longer present. MI interventions that include a focus on intrapersonal characteristics of teachers (e.g., self-efficacy, perceived competence, attitudes) could lead to more effective, sustainable interventions in the future [97].

### Delivery

It was evident in our review that an optimal delivery of MI has not been identified in the existing literature. The dose (frequency and duration), intensity, and type of physical activities varied substantially from study to study making it hard to make comparisons among studies (Table 2). As the results showed, student-driven interventions were focused mainly on light intensity of physical activity of no specific duration, evident throughout the school day. On the contrary, the focus on MVPA was predominant in researcher-driven and researcher-teacher collaboration MI interventions. In teacher-driven interventions, an equal number of studies focused on MVPA (9 out of 15) and fundamental motor skills (8 out of 15). We did not identify substantial differences on the daily duration of the MI activities across different designs (with the exception of the student-driven interventions). The most common duration was between 10 and 20 min per day with some exceptions being as low as 3–4 min per day (activity break of high intensity) or as high as 50–60 min (full lesson plan integrated with academics). It was interesting that the researcher-driven interventions recommended MI on a daily basis, whereas most teacher-driven and researcher-teacher collaboration interventions provided more flexibility with a 2–5 days per week implementation. It is possible that a different duration, frequency, intensity, or type of MI is needed when the activities are integrated with academic subjects (such as math, language arts, science, and social studies), are activity breaks, or are offered as part of transitions, rewards, or awareness. Systematic and extensive research is needed in order to understand the optimal conditions for the delivery of MI with different characteristics and for different purposes.

Another area that needs extensive work is treatment fidelity. Treatment fidelity is the final stage in implementing an intervention and is defined as “strategies that monitor and enhance the accuracy and consistency of an intervention to ensure it is implemented as planned and that each component is delivered in a comparable manner to all study participants over time” (page 122, [101]). Even though over half of the interventions (49 studies) measured fidelity of implementation, of those, fewer interventions (35 studies) provided some information about whether the intervention was delivered as intended with the majority focusing only on the accumulation of minutes of physical activity but not on the qualitative characteristics of the program implementation (e.g., how it was perceived by teachers and students, whether modifications were necessary, and if yes, what modifications were made, were the expectations for the activities realistic and easy to conduct, etc.). Generally, teacher self-reports were used to measure fidelity of implementation, and in many cases, the data were collected after the completion of the intervention making the information less precise or accurate. This is an issue not only within MI intervention research, but also across other types of PA interventions [102]. It is recommended that studies report the fidelity of the implementation to the intervention protocol and the theoretical approach that was used (if any) [103, 104].

Overall, the information reported about the intervention in each study differs dramatically in its scope and depth. Some degree of reporting variance is to be expected, given that the studies were published in a wide range of journals that have different aims, target audiences, and submission requirements. In addition, there is considerable heterogeneity in the amount of the reported detail, given that in some interventions, MI is the sole independent variable, whereas in others, it is only one component of a multi-component intervention. However, a minimum standard for reporting MI intervention details should be established and followed to ensure that interventions can be carefully compared and replicated. For example, a recent study developed a 12-item checklist that can guide future reporting of MI interventions [105]. The items include the following: (a) brief name of the intervention; (b) rationale for the intervention; (c) materials used in intervention; (d) procedures used in intervention; (e) who provided the intervention; (f) modes of intervention delivery; (g) types of locations where the intervention occurred; (h) when and how the intervention was delivered (dose); (i) if the intervention was personalized and adapted and why; (j) modifications during the course of the intervention; (k) assessment of intervention adherence/fidelity; and (l) the extent to which the intervention was delivered as planned.

## Summary, Limitations, and Conclusions

This study presents a typology of MI interventions to highlight the different types of previous MI interventions in the primary/elementary classroom setting. The systematic review included a total of 72 MI interventions with variability in the approach, methodology, and scope of the study. Based on the results, we identified MI interventions with a different design, strategies, support, and delivery and qualitatively analyzed them to develop a typology and provide a review of the various intervention approaches that have been used in previous studies. Based on the results, it is clear that there are distinct approaches that are most often employed to promote the use of MI, and these approaches may often lack certain emphases, which could potentially enhance the effectiveness and sustainability of MI programming. It is possible that various aspects of the interventions reviewed in this study were not reported but were in fact implemented. Moving forward, it is important that authors strive to report as much detail about their MI interventions as possible in order to increase the transparency of these efforts. This will allow others who are interested in maximizing the effectiveness and sustainability of MI interventions to make more informed decisions about adopting, adapting, and creating MI programs that are best suited to specific contexts.

A limitation of this systematic literature review is that it did not evaluate the quality of the MI interventions or their effectiveness; therefore, no conclusions can be made regarding the feasibility and sustainability of the different characteristics identified in the typology to increasing MI. This approach was beyond the scope of this review in order to avoid the risk of eliminating a large number of MI interventions that may had adopted a more pragmatic approach in implementing physical activity in the elementary classroom. As with any systematic reviews, it is possible that MI studies conducted during the search period were missed to be identified by the researchers and included in this review.

To conclude, this study makes a unique contribution to the literature as it is the first systematic review that has developed a typology of MI interventions in the primary/elementary classroom with the largest number of included MI studies. It is anticipated that the developed typology may provide insights to researchers and practitioners to expand limited studies regarding MI strategies and designs, build on support systems that maximize effectiveness, identify the best practices on delivery, and recommend new directions for future growth. All of these factors may play a substantial role with respect on how to successfully translate MI research into best practice in the elementary classroom and should be further examined in the future.

## Additional Files

**Additional file 1.** Table with all the information retrieved from the MI intervention studies included in the systematic review.

**Additional file 2.** Supplemental References*.

## Data Availability

All data generated or analyzed during this study are included in this published article (and its supplementary online support file).

## Abbreviations

MI: Movement integration
PA: Physical activity
