# Supplementary material for: A Systematic Review and Qualitative Synthesis Resulting in a Typology of Elementary Classroom Movement Integration Interventions
Source: Sports Med Open. 2020 Jan 6;6:1. doi: 10.1186/s40798-019-0218-8 (PMC6944721; doi:10.1186/s40798-019-0218-8)
Supplement: Supplementary file 1 — Additional file 1. Table with all the information retrieved from the MI intervention studies included in the systematic review. [file 40798_2019_218_MOESM1_ESM.pdf]

Online Resourse: All information retrieved from the MI intervention studies included in the systematic review

Number of study	Authors	Country	Geographic Classifi-cation	Student Age; Grade Level	# of Students; Classes; Schools	Name of Program, Description	PA Characteristics	Intervention Dose	Instruction Type	Equipment/ Resources	Training	Fidelity (measure)	Fidelity (delivered as intented)	MI strategy
**Student-Driven**														
15	Benden, Blake, Wendel, & Huber (2011)	U.S.A.	Not Reported	Not Reported; 1st grade	N= 58 (31 T, 27 C); 4 Cls; 1 S	No official program name reported. Intervention classrooms were providecd with standing desks and standing height stools.	Students were informed that they could stand or sit at their discretion throughout the school day (Light)	Throughout the school day for a school year.	Student-led	Standing desks, standing height stools	Not provided	Not Reported	Not Reported	Physical Environment
16	Benden, Zhao, Jeffrey, Wendel, & Blake (2014)	U.S.A.	Not Reported	8.45 years T; 8.49 years C; 2nd-4th grades	N = 374; 24 Cls (12 T); 3 S	No offical program name reported. Stand-biased desks were provided to intervention classrooms to examine the effectiveness of standing workstations during lecture style teaching in math, history, and English.	Standing (Light)	Thoughout the school day for a school year.	Student-led	Stand-based desks	Not provided intentionally	Not Reported	Yes for intervention; Two control classrooms adopted an aletnative seating arrangement that did not serve as "business-as-usual" and were not considered in final analysis.	Physical Environment
17	Burgoyne & Ketcham (2015)	U.S.A.	Not Reported	Not reported; 2nd grade	N = 19; 1 Cls; 1 S; Within	No official program name reported. The classroom teacher was provided with therapy balls to be used as a substitute to standard chairs.	Dynamic sitting (Light)	Student seated behavior was observed once for 60 minutes in standard chairs and twice more when sitting on therapy balls. Acute effect.	Student-led	Therapy balls	Not reported	Direct observation by researchers	Not Reported	Physical Environment
18	Cardon, De Clercq, De Bourdeaudhuij, & Breithecker (2004)	Belgium	Not Reported	8 years old; M = 8.0-8.8; 2nd grade	N = 47 (22 T); 1 Cls; 1 S	**Moving School:** the school as a place of work. Classrooms were provided with ergonomic furniture and reorganized.	Static and dynamic sitting, standing, walking around, being active (like skipping, dancing, running), trunk flexion/rotation, neck flexion/rotation (Light)	Students were observed for 30 minutes during a lesson. Acute effect.	Student-led	Ergonomic furniture, tables with an inclinable top, stand-at desks	Not reported	A questionnaire about adjustment of the school furniture and observation of postures in the classroom	Yes. All children had furniture that were adjusted to their knee and elbow height.	Physical Environment
19	Contardo Ayala et al. (2016)	Australia	Urban	11.5 years T; 11.7 years C; Year 6	N = 48; 2 Cls; 1 S	**Make A Stand Kids (MASK)**; Each student in the intervention was provided with a manually adjustable height-adjustable workstation and a stool. Teachers received training/PD.	Light intensity active break from sitting (i.e., bean-bag throwing while spelling). (Light; Manipulative)	Extended blocks (60+ min) interrupted every 30 min with a 2-min guided light-intensity, a suggested 30-min standing lesson each day, and ergonomic furniture for standing for 8 months.	Student-led	Height-adjustable desks, stools	Yes. A professional development session was delivered to the teachers adapted largerly from the Transform--Us! Program. Included pedagogical approaches to reducing and breaking up sitting in the classroom, how to adapt the delivery of the curriculum and safety.	Accelerometers used only for PA behavior (sitting, standing, etc) during classroom time at two time points. Not full implementation measure.	Not Reported	Physical Environment; MB
20	Fedewa & Erwin (2011)	U.S.A.	Rural	9 years 11 months; 4th-5th grades	N = 76; 4 Cls; 1 S; Within	No official program name reported. Students were fitted for a stability ball using height measurements and used these balls as seats in the class.	Sitting on stability balls during academic lessons (Light)	Throughout the school day for 12 weeks	Student-led	Stability balls	Brief training to teachers provided by the physical education teacher and a researcher. No information about the content of training. Students were fitted for their stability balls by a trained representative from the company with the balls.	Direct observations throughout intervention periods (30 min , 3 times per week)	Yes	Physical Environment
21	Fedewa, Davis, & Ahn (2015)	U.S.A.	Rural	Not Reported; 2nd grade	N = 67 (36 T, 31 C); 4 Cls (2 T); 1 S	No official program name reported. Invention classrooms were provided with stability balls.	Dynamic sitting (Light)	Throughout the school day for a school year.	Student-led	Stability balls	Not reported	Direct observation by researchers	Not reported	Physical Environment
22	Harris et al. (1997)	USA	Urban and rural	4th - 5th grade	N = 171 (109 T); Not reported Cls; 4 S (2 T)	**Kansas LEAN** is a multi-component intervention with focus on nutrition and physical activity in classroom and in PE	Installed fitness station in classroom (e.g., stretch, sit & reach) and incentive system for its use (Fitness; Stretching)	1 academic year	Student-led	Not required	No training was provided for fitness stations in the classroom. On-site coordinators provided training on nutrition education.	Initially monthy feedback in meetings with partnership leadership and staff. End of year survey with school staff.	Not Clear. Cummulate number of changes are reported.	MB; Rewards
23	Hinckson et al. (2013)	New Zealand	Not Reported	10 years; 3rd-4th grade	N = 30 (23 I); 3 Cls (2 T); 2 S	No official program name reported. Standing workstations were provided to intervention classrooms.	Standing, sitting (Light)	Throughout the school day for 4 weeks	Student-led	Circular workstations with standing desks, exercise balls, bean bags, and mats were made available for children to sit when tired. Traditional desks were removed.	Not reported	Direct observations weekly and interviews	Yes for 4th grade. Perceived as a distraction for 3rd grade class due to limited participants within the classroom.	Physical Environment
24	Pangrazi, Beighle, Vehige, & Vack (2003)	U.S.A.	Not Reported	9.8 years; 4th grade	N = 606; Not Reported Cls; 35 S (10 +9 T)	**Promoting Lifestyle Activity for Youth (PLAY)**: Promotes moderate to vigorous physical activity for children in grades for to six. This process-oriented program shifts the focus from fitness and toward regular participation in daily physical activity through self-monitoring by students (taught in steps).	Walking and a variety (15) of games/activities with and w/out teachers (Step 2) and 30 minute self-directed PA outside of school (Step 3) (MVPA)	15 minutes a day at school for 3 weeks and 30 minutes a day for 8 weeks (total 12 weeks)	Teacher-led; Student-led	Game/activity cards	Teachers received training from county health coordinators who facilitate PLAY. Training included extra focus on avoiding false motivators such as incentives and public display of PA behavior. County health coordinators visited schools throughout implementation to monitor and support.	Not Reported	Not Reported	MB
**Teacher-Driven**														
25	Bershwinger & Brusseau (2013)	U.S.A.	Rural	9.2 years; 4th grade	N = 18; 1 Cls; 1 S; Within	No official program name reported. The teacher and class used in the study had already developed and implemented activity breaks into their classroom. Those activities were used for this study.	Jumping jacks, spelling jacks, walking breaks, outdoor/classroom games, etc. (Fitness)	5-15 minutes, 1-3 times a day, for 1 week	Teacher-led	Not Reported	No training. The teacher/students had already implemented activity breaks into the classroom, so they were part of their daily routine.	Not Reported	Not Reported	MB

Number of study	Authors	Country	Geographic Classifi-cation	Student Age; Grade Level	# of Students; Classes; Schools	Name of Program, Description	PA Characteristics	Intervention Dose	Instruction Type	Equipment/ Resources	Training	Fidelity (measure)	Fidelity (delivered as intented)	MI strategy
26	Burns, Brusseau, & Hannon (2015)	U.S.A.	Not Reported	M = 9.6; 4th-5th grade	n = 327; Not Reported; 4 S; Within	This study utilized a Comprehensive School Physical Activity Program with main focus on PE. Multicomponent (recess, PE, classroom).	Stretching or relaxation break, walking around the classroom or hallway, jumping with an invisible jump rope, doing squats, push-up, or sit-ups, or passing a ball around the classroom (MVPA; FMS; Fitness/Resistance; Stretching)	10-15 minutes (2 or 3 times x 5-minutes) per day for 6 months.	Teacher-led	Not required	Not reported	Not Reported	Not Reported	MB
27	Carlson et al. (2015)	U.S.A.	Not Reported	8.8 years ± 1.5; 1-6 grade	N = 1322; 97 Cls; 24 S	No official program name reported. The California Endowment funded school districts to implement interventions to incorporate daily 10-minute physical activity breaks in the classroom using their choice of evidence-based programs.	Choice from a pool of evidence-based program such as Instant Recess, Take 10!, CATCH (MVPA)	10-minutes per day for a school year	Teacher-led	Materials specific to classroom physical activity breaks (e.g., handouts, instruction books, videos, websites)	A district-level coordinator was hired to provide teacher trainings, technical assistance, and support groups.	Teachers reported implementation. Implementation measure was not reported.	No; 64.7% and 70.1% of teacher (in two time points) held activity breaks ever	MB
28	Cluss et al. (2016)	U.S.A.	Rural	K-5th grade	N= average of 3027 students per year from 2006-2016; Not Reported Cls; 5 S; Longitudinal	**HEALTHY Armstrong initiativ**e is a multicomponent intervention including schools, community and parents, with goals to improve nutrition and increase PA.	Aerobic (jumping rope, jumping jacks), breakheart-pumping fitness stations, apple crunch activities, ways to have fun with different kinds of sports balls and other sports equipment. Brain Breaks. Teachers were encouraged to offer organized fitness activities as rewards for classroom behavior. (MVPA; Fitness)	Varied by school and year.	Teacher-led	A 50-page (10-lessons) manual of nutrition information and fitness activities. Jump rope, balls/sports equipment, inexpensive equipment	Teachers received a manual with lessons. A school-district coordinator shared information about creative programs developed in one school across all schools and provided support throughout the intervention. Wellness facilitators were teachers in each school and they participated in workshops each year.	Teacher self-reports, informant interviews, and monthly tracking log.	Yes	MB; Opening Activity; Reward
29	Erwin, Abel, Beighle, & Beets (2011)	U.S.A.	Not Reported	8-12 years; M = 10.07; 4th-5th grade	N = 75; 4 Cls; 1 S; Within	No official program name reported. Twenty three activiites were created or modified from existing resources to be included with math core content.	Hopping, jumping, walking, bending, twisting, curling, jumping jacks, lunges, and squats (MVPA; FMS)	10 minutes a day, 5 days a week, for 13 days.	Teacher-led	Not required	Teachers were provided with all-day in-service training to develop physical activities in the form of lesson plans.	Not Reported	Not Reported	Integrated: Math
30	Grillich, Kien, Takuya, Weber, & Gartlehner (2016)	Austria	Not Reported	N = 8-9 yearls old; Year 3 primary school	N = 925; 53 Cls (26 T); 45 S	**Classes in Motion** based on Healthy Lower Austria. Multicomponent intervention with improved quality PE and promotion of health-related behaviors, such as physical activity during the school day.	Not Reported	Not Reported	Teacher-led	Not Reported	20-hour tailored on-the-job training by a qualified health promotion specialist partly during the regular classes, covering topics such as active teaching, motivational techniques, and safety.	Not Reported	Not Reported	MB
31	Meyer et al. (2015)	Switzerland	Urban, rural	6-8 and 11-12 years; 1st and 5th grades	N = 502; 28 Cls; 15 S sample varies by publication	**Kinder- und Jugendsportstudie (KISS):** Multi-component intervention, with 2 additional PE lessons per week, activity breaks, adaptation of playgrounds, and PA homework.	Activity breaks were motor skills such as jumping around on one leg, balancing on one leg, power games or coordinative tasks. (FMS)	6-25 minutes per day (2-5 minutes, 3-5 times per day) for a school year	Teacher-led	Not Reported	Not reported	Not Reported	Not Reported	MB
32	Kvalø, Bru, Brønnick, & Dyrstad (2017)	Norway	Urban, suburban	9-10 years, 5th grade	N = 484 (251 T); Not Reported Cls; 9 (5 T) S	**Active School**: Multicomponent intervention that included PE, integrated lessons, activity breaks, PA homework, recess. Focused on improved quality, frequency and variety of PA.	Running forward/backward/in pairs, jump and hop on one foot in the classroom and playground. (FMS; MVPA)	45-minutes (Integrated) 2 times per week, 5 x 10 minutes (MB) per day,for a school year.	Teacher-led	Website; fintess equipment (jump ropes, slopes, and cubes, dice)	Teachers received training from research team before the implementation and throughout the school year (regular meetings). At midway, a workshop for all teachers was provided. A website was developed to enable lesson plan sharing between teachers. Teachers attended 3 PA courses during the school year on orienteering, dance, and Roris (teaching tool for MB).	Not Reported	Not Reported	Integrated: Mathematics, Norwegian, English; MB
33	Liu et al. (2008)	China	Not Reported	6-12 years; 1-5 grade	N = 753 (328 T); 26 Cls (14 T); 2 S	**Happy 10**: program based on the principles of TAKE 10!	Included many safe and age- and space-appropriate physical activities based on TAKE 10! program. No further information was provided. (MVPA)	10 minutes per day for 8 months.	Teacher-led	Not Reported	Not Reported	Tracking poster and stickers were used to illustrate progress. Physical activity monitors were used to assess the intensity of the sessions with a sub-sample.	Not Reported	Not Reported
34	Naylor et al. (2008)	Canada	Not Reported	9-11 years old; 4th-5th grade	N = 237 (156 T, 81 C); Not Reported Cls; 8 S (6 T, 2 C) sample varied per publication; Longitudinal	This study utilized a multicomponent program titled **Action Schools! - BC that included** classroom Action Zone for increased physical activity and Bounce at the Bell for short classroom activity breaks. PE time was increased in addition to movement breaks to meet the 150 minutes of PA per week.	Classroom Action Zone: Skipping, dancing, playground circuits, resistance exercises; Bounce at the Bell: counter-movement and side-to-side jumps, video dance clips (MVPA; Dance; FMS; Fitness/Resistance)	15 minutes per day (with goal 75 minutes per week) for 16 months.	Teacher-led	Classroom Action Bin with resources: playground balls, videos, skipping ropes, exercise bands, bean bags, strength grippers, written material, school newsletters for families.	Intervention teachers were given a 1-day training workshop from the support team. School facilitators (2 PE teachers) provided support to schools on a weekly basis. Ongoing consultation (onsite and telephone) was provided to administrators and teachers. Liason and champion schools had different level of support.	Teacher-reported activity logs (type, frequency, duration of activity in the class)	Yes. Compiance was 67% (April-June) and 83% (Oct-May)	MB
35	Reed et al. (2010)	U.S.A.	Not Reported	9 -11 years; 3rd grade	N = 155 (80 T); 6 Cls (3 T); 1 S	No official program name reported. Intervention group integrated fundamental skills into their core curricula during integrative lessons.	Running, hopping, walking, skipping (FMS)	30 minutes a day, 3 times a week, for 4 months	Teacher-led	Not required	Teachers received two training sessions (about 90 min each) before implementation and 2 additional training sessions during implementation with focus on how to integrate PA with academics.	Direct observations of random lessons	Not Reported	Integrated

Number of study	Authors	Country	Geographic Classifi-cation	Student Age; Grade Level	# of Students; Classes; Schools	Name of Program, Description	PA Characteristics	Intervention Dose	Instruction Type	Equipment/ Resources	Training	Fidelity (measure)	Fidelity (delivered as intented)	MI strategy
36	Riley, Lubans, Holmes, & Morgan (2016)	Australia	Not Reported	11.1 years; 4th-5th grades	N = 240 (142 T); 10 Cls; 8 S	**Easy Minds Program:** Classroom teachers adapted mathematics lessons so that students were involved in movement-based learning.	Skipping, throwing and catching a ball or running through drill ladders, dancing (Macarena), slap count, aerobic routines. (FMS; Dance; Fitness/Aerobic)	60 minutes, 3 times per week, for 6 weeks	Teacher-led	Stopwatches, tape measures, balls, markers, and example activities developed by research team.	One day (6-hour) teacher professional learning workshop delivered by research team before program implementation. The workshop was credited. Email support for teachers was provided during program implementation.	Teachers completed a daily evaluation log. Observations (fidelity check) were conducted during weeks 1, 3, 5 of intervention. Student satisfaction was assessed with questionnaires upon completion of the program. PA during mathematics was measured throughout intervention.	Yes.	Integrated: Math
37	Sibley, Ward, Yazvac, Zullig, & Potteiger (2008)	U.S.A.	Not Reported	Not Reported	Not Reported; Not Reported; N=1	**Making the Grade with Diet and Exercise**: Multicomponent intervention including environmental chage to increase access to physical activity every morning, free breakfast, and reversal of order of lunch time with recess.	Walking, running, exercise videos, calisthenics, resistance training, and gymnasium and playground games (MVPA; FMS; Fitness/Resistance)	10-20 minutes at the beginning of each school day, for a school year.	Teacher-led	Exercise videos, possibly resistance training and/or gymnasium and playground game equipment	Not reported	Not Reported	Not Reported	MB; Opening Activity
38	Sirota et al (2014)	U.S.A.	Urban	10.0 years ± 0.68; 5th grade	N = 290; Not reported Cls; 7 S; Within	**CHALK/HSHF** (Choosing Healthy & Active Lifestyles for Kids/Healthy Schools Healthy Families): Transition Exercises (TE) consist of multiple physical activity sessions. Exercises were tied to the common core state standards, allowing teachers to introduce topics in math, science, language, and Arts.	Each session includes activities such as yoga, dance, and stretching. (Yoga; Dance; Stretching)	2-3 minutes with no strict guidelines on how many activity breaks should be incorporated. Teachers implemented variable numbers of breaks on any given day.	Teacher-led	Not required	Teachers received training. No information about training.	Teachers completed weekly logs	Ten minutes per week reflects the mean number of minutes students participated in TE.	Integrated; Transition
39	Snyder et al. (2017)	U.S.A.	Not Reported	Not Reported; 3rd grade	N = 24 (11 T); 2 Cls (1 T); 1 S	No official name reported. Teachers developed purposeful movement teaching in mathematics.	Typical activities involved incorporating fitness exercises such as burpees, jumping jacks, and squats into the lesson. (MVPA; Fitness)	70 minutes per day for 5 weeks	Teacher led; PE teacher acted as fitness liaison.	Twenty five lessons developed by teachers and materials already available in classroom (whiteboards, dice, blocks).	Yes, by the PE teacher. When and what kind of training was not reported.	Not Reported	Reported that the intervention was conducted successfully but no measure or results were shared.	Integrated: Math
**Research-Teacher Collaboration**														
40	Donnelly et al. (2009);	U.S.A.	Not Reported	Not Reported; 2nd-5th grades	N = 4905; Not Reported Cls; 24 S(14 T)	**Physical Activity Across the Curriculum (PAAC):** Physically active lesson plans were designed to target cognitive and motor development of students.	Existing lessons from teachers and examples from Take 10! Activities (MVPA)	10 minutes, 90 minutes per week, for three school years.	Teacher-led	TAKE10! material kits, sample lessons provided in a notebook	Teachers received an one day (6-hour) workshop through in-service training at the beginning of the school year.	Weekly teacher log, online weekly teacher survey, direct observations, end of school year focus groups	Partially. Weekly classroom physical activity averages ranged form 45-75+ minutes per week.	Integrated: Math, Language Arts
41	Donnelly et al. (2017)	U.S.A.	Urban, rural	8.1 years; 2nd-3rd grade	N = 584 (316 T); Not Reported Cls; 17 S (9 T); Longitudinal	**Academic Achievement and Physical Activity Across the Curriculum intervention (A + PAAC)**: Integrates activity with academic instruction.	PAAC lessons integrate PA with academic instruction. (MVPA)	20-minutes per day (10 minutes x 2 times, one in the morning and one in the afternoon) for 3 school years.	Teacher-led	Written teachers guide, website with sample lessons, compensation	Teachers received two 6-hour in-service sessions conducted at the school by the research team before implementation with booster sessions (1-day, 4 hours) at the beginning of years 2 and 3.	Teachers completed logs; direct observation	Partially. Did not reach the goal of 100 minutes per week. On average 55 min/week of lessons were delivered.	Integrated
42	Dunn, Venturanza, Walsh, & Nonas (2012)	U.S.A.	Urban	Not Reported ; K-3rd grades	Not Reported ; 144 Cls (72 T); 39 S	**Move-To-Improve** integrated core academic requirements into fitness breaks.	Running, jumping, squatting, dancing, stretching, yoga, deep-breathing (MVPA; Dance; Yoga; Stretching)	9 minutes (3 minutes x 3 activities) per day. Acute effect.	Teacher-led	A manual detailing 30 fitness breaks, equipment kits including polyvinyl spot markers used to help children identify personal space, bean bags, scarves, 2 CDs, and a professional development stipend of $68.14.	Teachers attended one 3-hour workshop before implementation.	Direct observation	Yes (71/72 of the trained classroom teachers led their students in physical activity and averaged 9.5 minutes per day).	Integrated
43	Erwin, Beighle, Morgan, & Noland (2011b)	U.S.A.	Not Repored	M = 10.07; 3rd-5th grade	N = 106; 15 Cls (9 T); 2 S (1 T)	No official program name reported.	Walking, abdominal, locomotor, upper body strength, or flexibility exercises. Autonomy on frequency, duration, and type of activities was provided. (MVPA; Fitness)	5-10-minute per day. Duration of intervention is not provided.	Teacher-led	Activity break cards, CD, web resources	Teachers received two 30-minute training sessions by two experts on classroom-based PA. One training was before intervention and the second was a booster training 1 month later.	Teacher-reported activity logs (frequency of classroom physical activity breaks)	Partially. Five teachers met their goal and four teachers did not.	MB
44	Katz et al., (2010)	U.S.A.	Not Reported	Not Reported; 2-4 grades	N=1214 (655 T); not reported Cls; 5 S (3 T)	**Activity Burts in the Classroom (ABC) for Fitness:** the program was developed with input from experts in education and provides fun and creative activities that are noncompetitive, age-appropriate, and gender-neutral to promote an interest in physical activity.	Each activity had 3 components: 1) a warm-up with stretching or light aerobic activity (eg, walking, arm circles, muscle stretching), 2) a core activity consisting of strength activities or aerobic activities (e.g., hopscotch, lunges, squats, star jumps, jogging), and 3) a cool-down similar to warm-up activities. Teachers were instructed to use all 3 components, but they were not required to offer a particular ratio of strength activities to aerobic activities (MVPA; Fitness)	30 minutes (multiple breaf sessions) per day for 9 months	Teacher-led	ABC fitness manual	Training sessions were provided to teachers before the start of the school year. No further information provided.	Not Reported	Not Reported	MB

Number of study	Authors	Country	Geographic Classifi-cation	Student Age; Grade Level	# of Students; Classes; Schools	Name of Program, Description	PA Characteristics	Intervention Dose	Instruction Type	Equipment/ Resources	Training	Fidelity (measure)	Fidelity (delivered as intented)	MI strategy
45	Martin & Murtagh (2017)	Ireland	Not Reported	8.9 years; 3rd-5th grades	N = 248; 10 Cls; 10 S	**Active Classrooms:** Developed to educate, train, and enable primary teachers to change their teaching methods toward engaging children in physical activity while learning academic content.	Active learning by demonstrating moves in stories (e.g., skip, run, hop), activity cards with exercises such as jumping, high knees etc. (MVPA)	20 minutes (2 x 10 minutes, one with Math and one with English) for 8 weeks.	Teacher-led	Lessons plans created by research team (20 English and 20 Math) and resources to teach them.	Teachers received an one-to-one 30 minutes training with the researcher with ideas on how to integrate PA into the lessons. Support throughout implementation.	Daily Teacher log, accelerometer data, focus group discussion and surveys at the end of intervention	Yes. High satisfaction with lessons.	Integrated: Math, English
46	Oliver, Schofield, & McEvoy (2006)	New Zealand	Urban	8- 10 years; 5th-6th grade	N = 78; 3 Cls; 1 S; Within	No official program name reported. A unit was developed in collaboration with primary school teachers (before intervention). All disciplines were linked by a common topic of conducting a ''virtual'' walk around New Zealand.	Walking and practicing various physical activities (e.g., running around 2 chairs; throwing a ball into a bin).	Four weeks. No further information is provided.	Teacher-led	Lesson plans	Teachers were provided an explanation of the resource upon delivery by a trained researcher before treatment.	Not Reported	Not Reported	Integrated English, social studies, mathematics, statistics, and physical education
47	Resaland et al. (2016)	Norway	Not Reported	10.2 years; 5th grade	N = 1129 (596 T); Not Reported Cls; 57 S	**Active Smarter Kids (ASK)**: Multicomponent intervention comprised three components (integrated lessons, activity breaks, PA homework) aimed at providing children with the opportunity to engage in 165 min of PA/week more than the control group.	Choice from a pool of physical activities (i.e., running, relay racing, obstacle courses, various forms of high-activity play, etc.). Goal for 25% of daily PA to be vigorous. Integrated lessons were mainly carried out in the playground and activity breaks in the classroom. (MVPA)	30 minutes a day for 3 days a week (Integrated lessons) and 5 minutes a day for 5 days a week (MB), for 7 months	Teacher-led	A website with information, videos, and content of 100 PA lessons. Laminating machines/accessories, mathematics bingo tiles, cones	Teachers received 3 seminars by the research team before the intervention and 2 refreshing sessions during the intervention period. Support via email and phone was also provided throughtout.	Teacher completed a log each month	Yes. Teacher-reports of PA indicated high adherence to the intervention and a clear contrast between the groups.	Integrated: Norwegian, mathematics, English; MB
48	Vazou & Skrade (2017)	U.S.A.	Rural	Not reported; 4th- 5th grade	N = 284 (157 T); 14 (7 T); 4	**Move for Thought**: Ten examples of how to integrate physical activity with academic subjects in the classroom with several variations and tips for teachers to modify the activities and adapt them based on their needs.	Developmentally appropriate fundamental motor skills (mainly locomotor skills, like jumping, skipping, animal-like walking), balancing, games with balls, and group challenges in the classroom. (FMS; MVPA)	10 min per day for 8 weeks	Teacher-led	Ten movement activity cards with games.	No training.	Teachers completed daily logs	Partially. Implemented on average 50% (±10%) of the days school was in session, with average duration of an activity being 10 (±1.5) min, resulting in 15 to 30 min of PA per week.	Integrated: Math
49	Whitt-Glover, Ham, & Yancey (2011)	U.S.A.	Not Reported	3rd-5th grade	N = 4,599; Not Reported Cls; 8 S (7 I)	**Instant Recess:** Provide opportunities for students to participate in 10-minute physical activity breaks, anywhere, anytime, in any type of attire.	Basic aerobic dance, calisthenics, sports movement set to music (MVPA)	10 minutes a day as a whole school (as desired), and 10 minutes a day at the teachers discretion for 8 weeks	Teacher-led; CD-led	CD or DVD	8 school staff representatives (teachers and at-least one school administrator) received a 3-hour training workshop led by the researchers before implementation.	Direct observation and a summative teacher log	Partially. Teacher logs ranged from 0 to 6 breaks per day. Classrooms that were observed had 8-10 minutes of PA but the activities were not always from the program.	MB
**Research-Driven**														
50	Anderson et al. (2016);	England	Urban, rural	9.5 years old; Years 4-5	N = 2221 (1064 T); Not Reported Cls; 60 S (30 T)	**Active for Life Year 5 (AFLY5)**: Multicomponent intervention aimed to increase children's self efficacy and knowledge, together with motivating parents, to increase children's levels of PA, reduce sedentary behaviour, and increase consumption of fruit and vegetables, through lessons and homework plans.	Games based on the food groups. No further information provided.	No information provided. Intervention lasted 12 months.	Teacher-led	16 lesson plans (on physical activity, nutrition or screen time), teaching materials, pictures, CDs, journals	Teachers received a full-day (8-9 hours) of training by the trial manager, a nutritionist, and a PE specialist.	Direct observation, teacher logs, and interviews with teachers and focus groups with children at the end of intervention	Yes	Integrated: Nutrition
51	Bailey & DiPerna (2015)	U.S.A.	Rural	Not Reported; 1st-2nd grades	N= 90; 6 Cls; 1 S; Within	**LEAP** proggram that provided **Energizers** activities	Not provided.	10-20 minutes twice a day, for 5, 7, or 9 weeks	Teacher-led	Packet of Energizers	A 2-hour training session beore implementation and weekly consultation sessions with the researchers throughout the implementation.	Direct observation and weekly teacher log	Yes	Integrated
52	Colin-Ramirez et al. (2010)	Mexico	Urban	8-10 years; 4th-5th grade	N = 498 (245 T); Not Reported Cls; 10 S (5 T)	**RESCATE:** Multicomponent intervention with focus on PE, activity breaks in classroom, health education, and take-home activities. A Take10! procedure manual was provided to the teachers.	Take10! Program (MVPA)	2–10 minutes per day for 12 months.	Teacher-led	Take10! manual	No training for activity breaks. Only for health lessons.	Not reported	Not reported	Integrated
53	Drummy et al. (2016)	Northern Ireland	Not Reported	9-10 years old, M = 9.5; Not Reported	N = 120; 14 Cls (7 T); 7 S	No official program name reported. An activity package was given to schools with encouragement to vary the activities each day.	Gentle jogging on the spot as warm-up for less than 1 min, followed by moderate-vigorous intensity exercises, such as hopping, jumping, running on the spot, scissor kicks, etc. beside the desks. (MVPA)	15 minutes (5 minutes 3 times per day), for 12 weeks.	Teacher-led	Information packs with detailed instructions for approximately 40 activity breaks.	Teachers and principals met with research team before implementation and received information packs on the activity breaks	Not Reported	Not Reported	MB
54	Erwin, Fedewa, & Ahn (2012)	U.S.A.	Not Reported	M = 8.87; 3rd grade	N = 29; 2 Cls (1 T); 1 S	No official program name reported. The 'Promoting PA & Health in the Classroom' (2009) book was utilized.	Promoting Physical Activity and Health in the Classroom activity break cards (MVPA)	20+ minutes per day for 20 weeks.	Teacher-led	Activity break cards, CD, web resources	The teacher was provided with a 30-minute classroom training before the intervention by an expert in classroom-based PA. The teacher also had previous training on physical activity promotion with youth (two college courses).	Teacher-reported activity log (name and nature of the break, duration, time period)	Not Reported	Integrated: math, reading
55	Evans et al., (2016)	USA	Not Reported	N= 7-11 years old; 3rd grade	N=1326; Not reported; 26	**Texas Grow! Eat! Go! (TGEG)** is a multicomponent program on school health using the CATCH program and including during school, after school programs, and parent involvement.	30 classroom PAs integrated with academics. No information is provided on the activities.	8 weeks in the fall and 6 weeks in the spring. No further information on dose.	Teacher-led	CATCH Kits, Walk Across Texas materials	Schools received a training session on CATCH before the beginning of the school year.	Teacher logs and questionnaires	Partially (M = 56% student participation). Varied by schools, ranging from 24 % to 90 % .	Integrated

Number of study	Authors	Country	Geographic Classifi-cation	Student Age; Grade Level	# of Students; Classes; Schools	Name of Program, Description	PA Characteristics	Intervention Dose	Instruction Type	Equipment/ Resources	Training	Fidelity (measure)	Fidelity (delivered as intented)	MI strategy
56	Fedewa, Ahn, Erwin, & Davis (2015);	U.S.A.	Urban	Not Reported; 3rd-5th grade	N = 460 (156 T); 38 Cls (15 T); 4 S	No Official Program Name reported. Teachers utlized standardized movement cards to integrate movement into their core academic curricula.	Aerobically-based activities that were developmentally appropriate (e.g., the teacher calls out a letter, color, or number and students move around the room trying to find the designated card). (MVPA)	20 minutes a day for 8 months.	Teacher-led	Not required	Two training sessions related to the PA break cards led by the researchers.	Daily PA logs, drop-in observations by student researchers, pedometers	It is uncertain as teachers did not complete their logs consistently.	Integrated: Math, Language Arts
57	Geldhof et al., (2006)	Beligium	Not Reported	M= 11.3 years; Not Reported	N= 365 (193 T); 20 Cls (10 T); 8 S (4 T)	No official name reported. A multi-factorial intervention consisted of a back education program and the stimulation of postural dynamism (frequent posture changes in addition to variable and dynamical activities) in the classroom.	Stimulating dynamic sitting, active and variable sitting were reinfroced with two prezi balls and a dynair and a wedge in each clasroom, coupled with two movement breaks a day. (Light)	2 short breaks per day for 2 school years	Teacher-led; Specialist- led (physical-therapist-led)	Prezi balls, dynair, wedge, manual with lesson and back pain information.	Teachers attended the back education lessons and the postural dynamism lessons delivered in the classroom.	Portable Ergonomic Observation method	Not Reported	Awareness
58	Goh et al. (2014)	U.S.A.	Not Reported	8-12 years; 3rd-5th grade	N = 210; 9 Cls; 1 S; Within	This study utilized a program titled **Take10!**.	Take10! Program (MVPA)	10 minutes per day for 12 weeks.	Teacher-led	Take10! Materials	Teachers attended a 1-hour training before the intervention and were given consultation support by the researchers throughout the implentation.	A weekly log with frequency of activities.	Yes	Integrated
59	Goh, Hannon, Webster, Podlog, & Newton (2016)	U.S.A.	Urban	8-12 years; 3rd-5th grades	N = 210; 9 Cls; 1 S; Within	**Take 10!:** This intervention consisted of a variety of 10-minute activities integrated with health and nutrition.	Take10! Program that includes an exercise, a cool down period, and a series of questions related to health and nutrition. T (MVPA)	10-minutes per day for 8 weeks	Teacher-led	Website with access to the Take 10! Program	Teachers received an 1-hour training by the researcher before implementing the program.	Direct observations and teacher logs	Not Reported	Integrated
60	Gortmaker et al. (1999)	USA	Not Reported	4th - 5th grade	N = 2103 (190 T cohort; 173 T subsample); Not reported Cls; 14 S (6 T); Longitudinal	**Eat Well & Keep Moving** is a multicomponent intervention focused on nutrition, screen time, and physical activity in the classroom, in integration with academics.	Classroom lessons with a physical education theme involving students in movement. No further information provided.	3 50-minute lessons per week for a school year	Teacher-led	Not Reported	Classroom teachers attended 1 day training and 2 staff wellness meetings each year.	Surveys of teachers after lessons were implemented.	Not Reported	Integrated
61	Grieco, Jowers, & Bartholomew (2009)	U.S.A.	Suburban	3rd grade	N = 97; 9 Cls; 1 S; Within	The **Texas I-CAN** program was designed to achieve academic curriculum goals through movement in the regular education classroom.	Movement in classroom of MVPA. Activities were similar to Take 10! Program (MVPA)	10-15 minutes per day on 4 of 5 days per week for a school year.	Teacher-led	Curriculum of lessons	Teachers received a full day of training (8 h) by the researchers on the implementation of the lessons before the implementation and a booster training midway through the school year.	Direct observation and daily teacher log (lesson time, duration, quality, ease, enjoyment etc).	Yes. Actual implementation was higher (91.5%)	Integrated
62	Grieco, Jowers, Errisuriz, & Bartholomew (2016)	U.S.A.	Suburban	M = 9.5 years; 3rd-5th grade	N = 320 (157 T); 20 Cls (10 T); Not Reported S	No official program name reported. Experiment. Students participated in one of 4 conditions (sedentary non-competitive lesson, sedentary competitive game, low-moderate PA competitive game, MVPA competitive game).	"Spelling relay" lessons of low-moderate and moderate-vigorous PAs. Walking, running, star jumps in a competitive game (low-moderate, MVPA)	10–15 minutes. Acute effect.	Researcher-led	Not required	Delivered by the researcher	Physical activity with accelerometers.	Yes	Integrated: language arts
63	Hill et al. (2010)	Scotland	Not Reported	8-11 years; 4th - 7th grades	N = 1224; Not Reported Cls; 6 S; Within	The **Classroom Exercise Program (CEP)** was developed by the Curriculum Support team for Physical Education, Health and Wellbeing in Aberdeen City Council and includes exercises while standing behind the desks .	Stretching and aerobic physical exercises (e.g. running on the spot, hopping sequences to music) behind the desks (MVPA)	10 -15 minutes per day (30 minutes after lunch) for 2 weeks	Teacher-led	Not Reported	Not reported	Not Reported	Not Reported	Not Reported
64	Hill, Williams, Aucott, Thomson, & Mon-Williams (2011)	Scotland	Not Reported	8-12 years, M= 9.8 years	N = 552; Not Reported Cls; 9 S; Within	No official program name reported. Exercise intervention developed by Aberdeen City Council physical education curriculum support team. Directed by the teacher within the classroom.	Students completed exercises while standing behind their desks. Jogging on the spot, basic sequences of jumps, etc. (MVPA)	10-15 minutes per day for 1 week. Acute effect.	Teacher-led	Not Reported	Not reported	Not Reported	Not Reported	MB
65	Howie, Schatz, & Pate (2015)	U.S.A.	Not reported	9-12 years; 4th-5th grades	N = 96; 5 Cls; 1 S; Within	**Brain Bites** is a simple classroom exercise break intervention designed to maintain moderate-to-vigourous physical acitivty during the movement break.	Activities performed with music in minimal space including stationary marching with arm movements and various forms of jumping and running in place. The breaks started with 30 seconds of low physical acitivty, and ended with a brief breathing acitvity. (MVPA)	Each session time varied between 5, 10, and 20 minutes. Acute effect.	Researcher-led	Not required	Delivered by the researcher	Direct observation	Yes	MB
66	**Hunter,** Abbott, Macdonald, Ziviani, & Cuskelly (2014)	Australia	Not Repoted	10 years; Year 5	N = 107 (55 T); 4 Cls (2 T); 1 S	This study utilized a program titled **Active Kids Active Minds (AKAM),** that implemented a preexisting 30 min **Smart Moves** program plus 30 min of PA during curriculum.	Running/brisk walking, running games, continuous relays (MVPA)	60 minutes per day for approximately 20 weeks.	Physical education teacher-led	Not Reported	A qualified Physical Education teacher was hired to run the intervention.	Direct observation, teacher log, pedometers	Partially/ on average 20 out of 30 min of class time was at MVPA. Three out of the four classes (1 in T and 1 C) had a change to their main teacher for one of the two terms of the intervention time.	MB
67	Keihner et al. **(2016)**	U.S.A.	Urban	9.74 years; 4th-5th grades	N = 3463 (1571 T); Not Reported Cls; 44 S	**Power Play! Campaign** is a multicomponent intervention conducted during/after school, including weekly nutrition and PA lessons, PA breaks, taste test, home, and community settings.	Not Reported	10 weeks. No information on dose.	Teacher-led	Not Reported	Not reported	Not Reported	Not Reported	Not Reported

Number of study	Authors	Country	Geographic Classifi-cation	Student Age; Grade Level	# of Students; Classes; Schools	Name of Program, Description	PA Characteristics	Intervention Dose	Instruction Type	Equipment/ Resources	Training	Fidelity (measure)	Fidelity (delivered as intented)	MI strategy
68	Ma, Mare, & Gurd (2014)	Canada	Not Reported	Not Reported; 2nd and 4th grade	N = 50; 2 Cls; 2 S; Within	**FUNtervals**: A 4 min high-intensity interval protocol that incorporated activity breaks in the classroom.	Consists of 20 s of high-intensity activity separated by 10 s of rest, repeated 8 times. Movements targeted included squats, jumping jacks, scissor kicks, jumping, and running on the spot. (MVPA)	4 min high intensity activity break (within a 10 minute break) on alternating days for 3 weeks. Acute effect.	Not reported	Not required	Not reported	Direct Observations for last 2 weeks	Yes; 5 activity breaks for 2nd and 4th grade (alternat school days for 2 weeks)	MB
69	Madsen et al. (2015)	U.S.A.	Urban	Not Reported; 3rd-5th grade	N = 879; Not Reported Cls; 6 (4T)	**Energy Balance for Kids with Play (EB4K with Play)**: Multicomponent, school-based obesity prevention intervention designed to improve the nutrition and physical activity environments of schools through integrated education lessons delivered by a registered dietitian (RD coach) and organized activities led by a Playworks coach. Includes parent/community components.	Organized play during in-school and class game time.	Twice per month intervention lasted for 2 school years.	Dietitian-led; Specialist-led	Not Reported	Each school had its own Playworks coach who delivered class game time.	Not Reported	Not Reported	Not Reported
70	Mahar et al. (2006)	U.S.A.	Not Reported	Not Reported; K-4th grade	N = 243 (135 T); 15 Cls; 1 S	**Energizers:** Grade-appropriate phsyical activities integrated with academic subjects that involve no equipment and require little teacher preparation.	Approximately 10-min of integrated PA	10 minutes per day for 12 weeks	Teacher-led	Online resource of Energizers	A 45-minute training session before implementation that included background and practical experience	Physical activity with pedometers for one week	Yes	Integrated
71	McClary King & Ling (2015)	U.S.A.	Rural	M = 7.30- 8.56; K-3 grade	N= 999; Not Reported Cls; 4 S; No control; Longitudinal	No offical program name reported. Multicomponent intervention targeting nutrition and PA behaviors (strategies in PE, classroom, wellness policies, community).	Spark PE currilcum was used, one-shot health education classes were provided, and classroom teachers were given the TAKE 10! Manual. (MVPA)	10 minutes per day over two academic years.	Healthy Lifestyle Coaches-led	Take10! Manual	Trained Healthy Lifestyle Coaches were hired to implement the intervention	Program was implemented and overseen by three Healthy Lifestyle Coaches	Not reported	Integrated
72	McClelland, Pitt, & Stein (2015)	United Kingdom	Urban	7-13 years old; 2nd-8th grade	N= 348; Not Reported Cls; 10 S	**Move4words:** is based on embodied cognition and bodily awareness/mindful control with visual, motor, and auditory skills.	Visual attention and eye tracking skills, classroom mild aerobic exercise, concentrating on attention and limb movement patterns, cross-lateral body coordination, relaxation. (Light; Coordination; Concentration)	A 20-minute lesson daily for 12 weeks at the start of the school day during normal lessons.	Video-led; Teacher-led	DVD with short videos modeling activities. Detailed module with activity sessions.	Teachers received one 2.5-hour training session before implementation. Teachers were given a highly descriptive module with each element of the 60 daily activity sessions laid out step-by-step. Short video segments (45 sec to 2 min) gave the children instructions on how to perform each activity.	Not Reported	Not Reported	Awareness; Opening Activity
73	McKay et al (2005)	Canada	Not Reported	M age = 10.1 years; 4th - 5th grade	N = 124 (51 T); Not reported Cls; 3 S	**Bounce at the Bell**: Teachers instructed the children to perform 10 counter movement jumps, three times each school day (once at morning bell, once at noon bell, and once at home time bell).	10 counter-movement jumps (two foot take off, clutch knees, two foot landing) (MVPA)	3 minutes per day (10 counter-movement jumps, 36 total), for 8 months.	Teacher-led	Not required	A research assistant met the teachers 3 times throughout the school year to observe, demonstrate, and review the jumps.	Teacher log with a record of student attendance and the average number of jumps the class performed each day.	Partially; compliance ranged from 2 days per week to 5 days per week (M = 90±34 jumps per week meaning 3 days per week)	MB; Opening Activity; Transition
74	Mullender-Wijnsma et al. (2016)	Netherlands	Not Reported	M = 8.1; 2nd-3rd grades	N= 499 (249 T); 24 Cls; 12 S; Longitudinal	This study utilized a program titled **Fit en Vaardig op school (F&V)**.	Jogging, hopping in place, marching, jumping jacks (MVPA)	20-30 minutes (10-15 in math and 10-15 in language arts) 3 times per week, for 22 weeks per school year, for 2 school years.	Trained expert-led (1st year); Teacher-led (2nd year)	Whiteboard, PowerPoints, manual	In the first year, six primary school teachers were hired and trained to deliver the lessons. In the second year, the regular classroom teacher received training to deliver the lessons.	Direct Obersvation (6 days) and teacher log	Yes. The lesson time was always at or near the intended 20 to 30 minutes. Only 2 lessons were cancelled due to holidays.	Integrated: math, language arts
75	Murtagh, Mulvihill, & Markey (2013)	Ireland	Rural	9.3 ± 1.4 years; 2nd – 6th grade	N = 90 (39 T); 8 Cls; 4 S	**Bizzy Break!:** A 10-min activity break in the classroom.	Mobility activities & stretching with music, next to desk. (MVPA; Stretching)	10 miutes per day for 5 days	Teacher-led	Poster (summarizing the activities), teacher notes (with detailed information on the activities) and a music CD	No training provided	Teachers noted the time the activity break was conducted. Implementation measure was not reported.	Not Reported	MB
76	Norlander, Moas, & Archer (2005)	Sweden	Not reported	M age = 11.31 (SD = 1.09); 4th - 6th grade	N = 95 (84 T); 6 Cls; 2 S	No official program name reported. Treatment included a stretching and relaxation program focused on eliminating noise levels and stress in the classroom.	5-10 minutes program that included 3 groups of stretch exercises on upper body (arms, neck, back) that were repeated. The program ended with relaxation exercises (e.g., breathing) while seated. (Light; Stretching; Breathing)	5-10 minutes twice daily (immediately after morning break and after lunch) for 4 weeks.	Teacher-led	Not required	Not reported	Not Reported	Not Reported	Awareness
77	Peck, Kehle, Bray, & Theodore (2005)	U.S.A.	Suburban	6-10 years; 1st-3rd grade	N = 10; Not Reported; Not Reported; Within	This study utilized a program titled **Yoga Fitness for Kids**. School psychologist was provided with two yoga videotapes (3-6 age version, 7-12 age version).	Deep breathing, physical postures, relaxation exercises (Light; Flexibility; Concentration)	Children followed the yoga videotape 30 minutes, twice a week, for a period of 3 weeks.	Video-led	Videotape	Standardized video was used	Standardized videotape, treatment integrity checklist	Yes	Awareness
78	Piek et al. (2013)	Australia	Metropolitan and regional	4-6 years M = 5.42 years	N = 511; Not Reported Cls; 12 S (6 T)	**Animal Fun**: Provided 9 program modules aimed to develop motor and social skills, and increase students' confidence in their physical abilities by imitating the movements of animals in a fun, non-competitive way.	Imitating the movements of animals. Walking, jumping, hopping, skipping, throwing, catching, kicking. (FMS)	30-minutes a day, 4 times a week for 10 weeks	Teacher-led	Not Reported	Teachers received a one-day training course before impmplementation.	Direct observations and teacher logs	Not Reported	Not Reported

Number of study	Authors	Country	Geographic Classifi-cation	Student Age; Grade Level	# of Students; Classes; Schools	Name of Program, Description	PA Characteristics	Intervention Dose	Instruction Type	Equipment/ Resources	Training	Fidelity (measure)	Fidelity (delivered as intented)	MI strategy
79	Reznik, Wylie-Rosett, Kim, & Ozuah (2015)	U.S.A.	Urban	Intervention = 6.1 years; Control = 5.9 years; K-1st grades	N = 988 (500 T); 45 Cls; 4 S	**Children's Hospital at Motefiore Joining Academics and Movement** (CHAM): An audio CD which uses interval-based education-focused aerobic activities that teachers can implement by playing the CD in their classrooms.	It was based on the TAKE 10! Program concept and includes 25 lessons that were prerecorded. Each lesson has a 2 minute warm-up, a variable 6-minute aerobic activity, and a 2 minute cool down (MVPA)	30 minutes (3 x 10-minutes) per day for 8 weeks.	CD-led	Audio CD with guided lessons accompained by contemporary music	Not required	Direct observation, teacher logs, and teacher interviews at the end of implementation.	Partially. Daily lessons ranged from 0 to 4, M = 1.8 times per day.	Mathematics, phonics, grammar, geography, time, money concepts
80	Schmidt, Benzing, & Kamer (2016)	Switzerland	Not Reported	11-12 years M = 11.77 years; 5th grade	N = 92 (50 T); 5 Cls; 5 S;	Experiment. Students were separated into one of 4 groups (physical activity with high cognitive, sedentary with high cognitive, physical activity with low cognitive, sedentary with low cognitive) who participated in a 10 minute intervention.	Students in the combo groups had to touch numbers from 1 to 18 as quickly as possible, which had been randomly painted on the ground in a 5x5m area and keep repeating the task for 5 min. The physical group simply ran for 10 min at different speeds. (MVPA)	10 minutes. Acute effect.	Researcher-led	5x5m area with 1-18 painted on the ground, pencil and paper, a story	Delivered by the researcher	Heart rate monitors and self-reported perceived exertion.	Yes	MB
81	Stephen & Wentz (1998)	U.S.A.	Urban	8-10 years; 4th grade	N = 99 (45 T); 2 Cls (1 T); 2 S (1 T)	No official program name reported. Intervention program where teams of two medical students met with students for physical activity sessions in the classroom.	Repetitive movements of large muscle groups (MVPA; Fitness)	30- 35 minutes (5 minutes of warm-up, 20 minutes of continuous aerobic activity, 5-10 minute cool down), 3 times a week for 15 weeks	Medical-student led	Not Reported	Medical students received a 2-day orientation session to deliver the lessons.	Implemented by research team (medical students)	Yes	MB
82	Treu, Doughty, Reynolds, Nijke, & Katz (2017)	U.S.A.	Not Reported	8.7 years; 3rd grade	N = 1487 ; Not Reported Cls; 26 S (8 T1, 9 T2)	**Advancing School and Community Engagement Now for Disease Prevention (ASCEND)**: Multicomponent intervention included nutrition and brief bursts of physical in the classroom (i.e., ABC for Fitness). Enhanced intervention had nutrition and brief activity bursts plus a family/home component.	Activity bursts were designed to include a brief warm-up and cool-down (e.g. stretching or low-intensity activity) along with one or more core activities of higher intensity (e.g., jumping jacks, hopping in placed, or dancing to music) (MVPA)	30 minutes a day for a school year.	Teacher-led	ABC for Fitness manual, activity challenge cards, 3-minute sand timer, facilities for family night	Not reported	Not Reported	Not Reported	Not Reported
83	van den Berg et al. (2016)	Netherlands	Not Reported	10-13 years old; 5th-6th grade	N = 184; 8 Cls; 3 S; Within	No official program name reported. A movie of each session was provided to be shown in the classroom for students to follow and imitate. The movie was developed by researchers and two physical education teachers.	Interactive videos. Aerobic (jogging, jumping, pretending to swim), coordination (dancing, clapping), strength exercises (squats, abs, arm curls). (MVPA; Fitness/Aerobic; Fitness/Resistance; Dance)	A 12-minute exercise video shown once. Acute effect.	Video-led	DVD	Standardized video was used	Guided by researcher, standardized movie	Yes	MB
84	van Stralen et al. (2012)	Netherlands	Not Reported	9.8 years; 6th-7th grade	N = 600; Not Reported Cls; 19 S (9 T)	**JUMP-in Kids in Motion**: A multicomponent intervention primarily aimed at the promotion of sports participation and outdoor play. It targets PA by changing physical, social and political environmental determinants, and cognitive mediators. The **Class Moves** component focuses on activity breaks.	Calendars with recurrent breaks for PA, posture exercises during regular lessons (Light; MVPA)	8 months in year 1 and 9 months in year 2. The dose was not reported.	Not Reported	Not Reported	Not Reported	Not Reported	Not Reported	Not Reported
85	Vazou, Gavrilou, Mamalaki, Papanastasiou, & Sioumala (2012)	Greece	Urban; Rural	Not Reported; 4th-5th grade	N = 147; 15 Cls; 8 S; Within	No official program name reported. Pre-service classroom teachers developed and implemented classroom-based physical activities with academics.	Movement-based activities based on developmentally appropriate fundamental motor skills (e.g., skipping, galloping, and running). (FMS)	10 minutes for 6 days over 2 weeks . Acute effect.	Pre-service teacher-led	Small softballs	Yes. Pre-service teachers received training during college course before delivering the intervention.	Implemented by researcher (pre-service teachers)	Yes	Integrated: language arts, math, social studies
86	Wright et al. (2016)	U.S.A.	Urban, Suburban, Peri-urban	Not Reported; 3rd-4th grades	N = 1183; Not reported Cls; 24 S; Longitudinal	The **Fueling Learning through Exercise (FLEX)** is a multicomponent study with two programs, the **Just Move** with structured PA breaks with both high- and low-intensity movements and the **100 Mile Club** that encouraged children to walk, run, or wheel 100 miles over the course of the school year.	Just Move: jumping jacks, squats, stretches, yoga (Light, MVPA, Yoga)	5–15 minutes per day for 2 years	Teacher-led	Just Move: movement/activity cards	Teachers received training from research team before implementation and provided ongoing support throughout the interventio.	Program participation and attitudes by children at mid-point and post-intervention. Survey of teachers at baseline, mid-point and post-intervention. Direct observation once per year.	N/A Study protocol	Integrated

Notes: Cls = classes, S = School, T = treatment, C = control, MVPA = moderate to vigorous physical activity, FMS = fundamendal motor skills,
