# Supplementary material for: A Systematic Review and Qualitative Synthesis Resulting in a Typology of Elementary Classroom Movement Integration Interventions
Source: Sports Med Open. 2020 Jan 6;6:1. doi: 10.1186/s40798-019-0218-8 (PMC6944721; doi:10.1186/s40798-019-0218-8)
Supplement: Supplementary file 2 — Additional file 2. Supplemental References*. [file 40798_2019_218_MOESM2_ESM.pdf]

Supplemental References*

*Additional references used to retrieve information about the MI interventions included in the online resource

1. Ahamed Y, Macdonald H, Reed K, Naylor P, Lui-Ambrose T, McKay Y. Time devoted to physical activity does not compromise academic performance of elementary school children. Med Sci Sport Exer. 2006;38:S468.
2. Macdonald HM, Kontulainen SA, Khan KM, Mckay H. Is a school-based physical activity intervention effective for increasing tibial bone strength in boys and girls?. J Bone Miner Res. 2007;22:434-46.
3. Reed KA, Warburton D, Macdonald H, Naylor PJ, Mckay HA. Action Schools! BC: a school-based physical activity intervention designed to decrease cardiovascular disease risk factors in children. Prev Med. 2008;46:525-31.
4. Hartmann T, Zahner L, Pühse U, Puder JJ, Kriemler S. Effects of a school-based physical activity program on physical and psychosocial quality of life in elementary school children: a cluster-randomized trial. Pediatr Exerc Sci. 2010;22:511-22.
5. Zahner L, Puder JJ, Roth R, Schmid M, Guldimann R, Pühse U, Knöpfli M, Braun-Fahrländer C, Marti B, Kriemler S. A school-based physical activity program to improve health and fitness in children aged 6–13 years ("Kinder-Sportstudie KISS"): study design of a randomized controlled trial [ISRCTN15360785]. BMC Public Health. 2006;6:147.
6. Kriemler S, Zahner L, Schindler C, Meyer U, Hartmann T, Hebestreit H, Brunner-La Rocca HP, Van Mechelen W, Puder JJ. Effect of school based physical activity programme (KISS) on fitness and adiposity in primary schoolchildren: cluster randomised controlled trial. BMJ.2010;340:c785.
7. Meyer U, Romann M, Zahner L, Schindler C, Puder JJ, Kraenzlin M, Rizzoli R, Kriemler S. Effect of a general school-based physical activity intervention on bone mineral content and density: a cluster-randomized controlled trial. Bone. 2011;48:792-7.
8. Gibson CA, Smith BK, DuBose KD, Greene JL, Bailey BW, Williams SL, Ryan JJ, Schmelzle KH, Washburn RA, Sullivan DK, Mayo MS. Physical activity across the curriculum: year one process evaluation results. Int J Behav Nutr Phy. 2008;5:36.
9. Szabo-Reed AN, Willis EA, Lee J, Hillman CH, Washburn RA, Donnelly JE. Impact of three years of classroom physical activity bouts on time-on-task behavior. Med Sci Sport Exer. 2017;49:2343–50.
10. Martin R, Murtagh EM. Preliminary findings of active classrooms: an intervention to increase physical activity levels of primary school children during class time. Teach Teach Educ. 2015;52:113–27.
11. Campbell R, Rawlins E, Wells S, Kipping RR, Chittleborough CR, Peters TJ, Lawlor DA, Jago R. Intervention fidelity in a school-based diet and physical activity intervention in the UK: Active for Life Year 5. Int J Behav Nutr Phy. 2015:12:141.
12. Jago R, Rawlins E, Kipping RR, Wells S, Chittleborough C, Peters TJ, Mytton J, Lawlor DA, Campbell R. Lessons learned from the AFLY5 RCT process evaluation: implications for the design of physical activity and nutrition interventions in schools. BMC Public Health. 2015;15:946.

13. Kipping RR, Howe LD, Jago R, Campbell R, Wells S, Chittleborough CR, Mytton J, Noble SM, Peters TJ, Lawlor DA. Effect of intervention aimed at increasing physical activity, reducing sedentary behaviour, and increasing fruit and vegetable consumption in children: active for life year 5 (AFLY5) school based cluster randomised controlled trial. BMJ. 2014;348:g3256.
14. Mullender-Wijnsma MJ, Hartman E, de Greeff JW, Bosker RJ, Doolaard S, Visscher C. Improving academic performance of school-age children by physical activity in the classroom: 1-year program evaluation. J School Health. 2015;85:365-71.
15. De Greeff JW, Hartman E, Mullender-Wijnsma MJ, Bosker RJ, Doolaard S, Visscher C. Long-term effects of physically active academic lessons on physical fitness and executive functions in primary school children. Health Educ Res. 2016;31:185-94.
